# ^18^Fluorodeoxyglucose uptake in relation to fat fraction and R2* in atherosclerotic plaques, using PET/MRI: a pilot study

**DOI:** 10.1038/s41598-021-93605-x

**Published:** 2021-07-09

**Authors:** Elin Good, Miguel Ochoa-Figueroa, Magnus Ziegler, Marcus Ressner, Marcel Warntjes, Petter Dyverfeldt, Mark Lubberink, Håkan Ahlström, Ebo de Muinck

**Affiliations:** 1 Department of Health, Medicine and Caring Sciences, Linköping University, Linköping, Sweden; 2 Center for Medical Image Science and Visualization (CMIV), Linköping University, Linköping, Sweden; 3 Department of Cardiology, Linköping University Hospital, Linköping, Sweden; 4 Department of Clinical Physiology, Linköping University Hospital, Linköping, Sweden; 5 Department of Radiology, Linköping University Hospital, Linköping, Sweden; 6 SyntheticMR AB, Linköping, Sweden; 7 Department of Surgical Sciences, Section of Radiology, Uppsala University, Uppsala, Sweden; 8 Antaros Medical AB, Mölndal, Sweden

**Keywords:** Vascular diseases, Diagnostic markers, Imaging techniques

## Abstract

Inflammation inside Atherosclerotic plaques represents a major pathophysiological process driving plaques towards rupture. Pre-clinical studies suggest a relationship between lipid rich necrotic core, intraplaque hemorrhage and inflammation, not previously explored in patients. Therefore, we designed a pilot study to investigate the feasibility of assessing the relationship between these plaque features in a quantitative manner using PET/MRI. In 12 patients with high-grade carotid stenosis the extent of lipid rich necrotic core and intraplaque hemorrhage was quantified from fat and R2* maps acquired with a previously validated 4-point Dixon MRI sequence in a stand-alone MRI. PET/MRI was used to measure ^18^F-FDG uptake. T1-weighted images from both scanners were used for registration of the quantitative Dixon data with the PET images. The plaques were heterogenous with respect to their volumes and composition. The mean values for the group were as follows: fat fraction (FF) 0.17% (± 0.07), R2* 47.6 s^−1^ (± 10.9) and target-to-blood pool ratio (TBR) 1.49 (± 0.48). At group level the correlation between TBR and FF_mean_ was − 0.406, *p* 0.19 and for TBR and R2*_mean_ 0.259, *p* 0.42. The lack of correlation persisted when analysed on a patient-by-patient basis but the study was not powered to draw definitive conclusions. We show the feasibility of analysing the quantitative relationship between lipid rich necrotic cores, intraplaque haemorrhage and plaque inflammation. The ^18^F-FDG uptake for most patients was low. This may reflect the biological complexity of the plaques and technical aspects inherent to ^18^F-FDG measurements.

**Trial registration:** ISRCTN, ISRCTN30673005. Registered 05 January 2021, retrospectively registered.

## Introduction

Atherosclerosis is an inflammatory disease. Myocardial infarction and stroke are the predominant manifestations of this disease and are primarily caused by rupture of atherosclerotic plaque with subsequent arterial thrombosis. Mechanistically, inflammation inside atherosclerotic plaques has been proposed as the major pathophysiological process driving plaques towards rupture. Macrophages are the predominant inflammatory cell type inside atherosclerotic plaques^1^. Plaques with active inflammation are characterized by extensive macrophage accumulation and macrophages play a major role in the progression towards plaque rupture^2,3^. In addition, there are morphological plaque features that have been associated with plaque rupture. Among these features, *lipid* *rich* *necrotic* *cores* and *intraplaque* *hemorrhage* have been shown to increase macrophage infiltration and activation^4,5^. Thus, a link has been proposed between the degree of plaque inflammation and the extent of lipid rich necrotic core and intraplaque hemorrhage. The investigation of this relationship in patients would benefit from the application of quantitative imaging methods. Therefore, we performed a pilot study employing a novel, thoroughly validated quantitative MRI (qMRI) technique to measure the extent of lipid rich necrotic cores as well as intraplaque hemorrhage. To measure inflammation we quantified the uptake of ^18^F-fluoro-deoxy-glucose (^18^F-FDG) in the same plaques on images acquired using a simultaneous whole-body PET/MRI scanner^6^.

## Methods

### Patients

Patients with carotid plaque were selected for the study based on routine duplex ultrasound using criteria established for the European Carotid Surgery Trial. According to these criteria, a Doppler flow velocity ≥ 1.3 m/s at a Doppler angle of 50°–60° corresponds to a ≥ 50% stenosis^7^. Patients planned for endarterectomy were excluded, to enable clinical follow-up within the study. Other exclusion criteria were: > 80 years of age, previous carotid endarterectomy, carotid occlusion, diabetes mellitus, renal failure (glomerular filtration rate < 45 mL/min/1.73 m^2^), inflammatory diseases including malignancies, immunologic disorders, and treatment with immunosuppressive/anti-inflammatory agents. Following duplex ultrasound 12 patients underwent qMRI of the carotid artery in a 3T on-site, stand-alone MRI followed by off-site PET/MRI at a national imaging facility. Clinical characteristics, laboratory values, clinical events and medication were recorded for each patient on dedicated case record forms (Table 1).

**Table 1:** Baseline characteristics.

Variable	Results
**Gender, N (%)**	
Male	9 (75)
**Clinical data, mean (SD)**	
Age	73 (± 2.8)
BMI	27.4 (± 2.6)
**Location of plaque, N (%)**	
Right	7 (58.3)
Left	5 (41.7)
**Comorbidities, N (%)**	
Previous ischemic cerebrovascular event	9 (75.0)
Ischemic heart disease	6 (50.0)
Peripheral arterial disease	4 (33.3)
Atrial fibrillation	2 (16.7)
**Cardiovascular risk factors N (%)**	
LDL ≥ 1.8 mmol/L	2 (16.7)
Current smoking	1 (8.3)
Previous smoking	7 (58.3)
BMI ≥ 25	9 (75.0)
Hypertension	10 (83.3)
**Lab values, mean (SD)**	
Cholesterol (mmol/L)	3.4 (± 0.7)
HDL-cholesterol (mmol/L)	1.3 (± 0.40)
LDL-cholesterol (mmol/L)	1.6 (± 0.22)
HbA1c (mmol/mol)	33.5 (± 13.6)
hsCRP (mg/L)	1.9 (± 1.5)
**Medical treatment N (%)**	
Platelet inhibitors	11 (91.7)
Anti-coagulants	1 (8.3)
ACE-inhibitors	8 (66.7)
Calcium antagonists	4 (33.3)
Beta-blockers	6 (50.0)
Statin only	9 (75.0)
Statin + ezetimibe	2 (16.7)
Ezetimibe only	1 (8.3)

*ACE* angiotensin-converting-enzyme, *BMI* body mass index, *CRP* C-reactive protein, *GFR* glomerular filtration rate, *HDL* high-density lipoprotein, *hsCRP* high-sensitivity C-reactive protein, *LDL* low-density lipoprotein, *N* number of patients, *TC* total cholesterol.

The study was approved by the Swedish Ethical Review Authority (approval nr: 2017/545-31) and performed in accordance with the Declaration of Helsinki. Written informed consent was obtained from all study participants.

### Data acquisition

#### qMRI

3D qMRI was performed in a 3 T Ingenia scanner (Philips Healthcare, Best, the Netherlands) using an 8-channel carotid coil (Shanghai Chenguang Medical Technologies, Shanghai, China). High-resolution maps of the fat fraction (FF) and values of R2* relaxation rate per voxel were acquired applying an out-of-phase, in-phase, out-of-phase, in-phase scheme at echo times (T_e_) of multiples of 3.6 ms. The water-fat shift was maximized at 1.3 pixels and it was assumed that all voxels exhibited a single effective R2* relaxation. Proton density and T1 relaxation differences between water and fat were not taken into account. The signal magnitude S at each T_e_ was modelled according to:

$$S\left({T}_{e}\right)=\left[W+Fcos\left(\frac{2\pi {T}_{e}}{2.4}\right)\right]\times {e}^{\left(-{R}_{2}^{*}\times {T}_{e}\right)}.$$

W = water fraction, F = fat fraction. Water and fat percentages were calculated by division by the sum of the two. Other acquisition parameters were repetition time = 18 ms, turbo field echo factor 12 and flip angle 10°. Two regional saturation slabs of 80 mm were added inferior and superior to the acquisition volume, with a gap of 40 mm, to suppress signal from inflowing blood. We have previously validated this qMRI approach to quantify fat and R2* against 3D-histology showing strong correlations between FF and R2* versus lipid rich necrotic core and intraplaque hemorrhage, respectively^8^. Slice thickness for Dixon images was 0.7 mm, with in-plane resolution 0.60 × 0.60 m^2^. A T1W turbo-spin echo acquisition was performed with echo time = 9 ms, repetition time = 1 heartbeat, and turbo-spin echo factor 6. T1W images had slice thickness 1.75 mm, and in-plane resolution 0.50 × 0.50 mm^2^.

#### PET/ MRI

**Figure 1:**
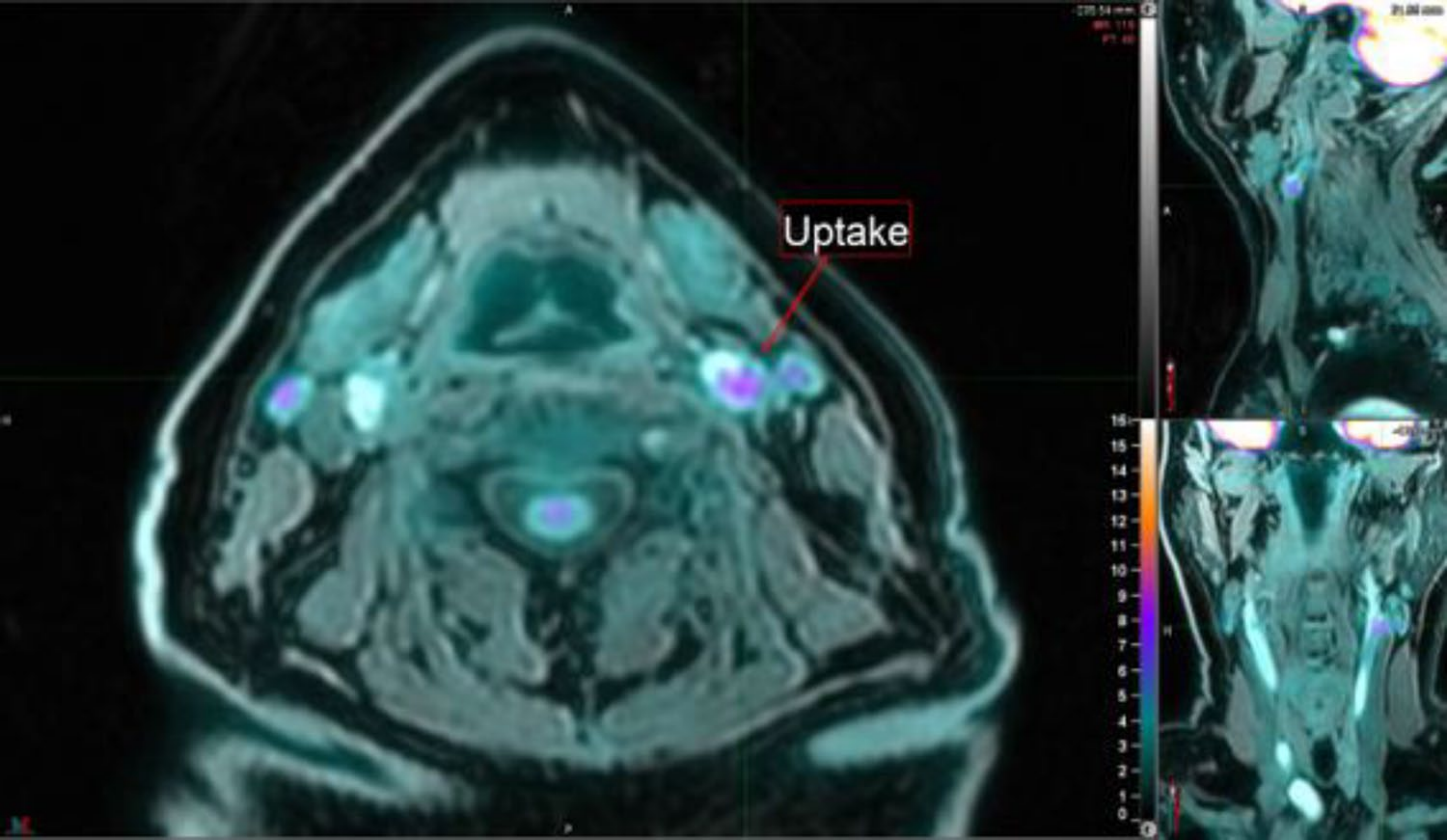
Increased ^18^F-FDG uptake in a carotid plaque (shown by the red arrow) in fusion images consisting of T1W MRI and PET. The anatomy of the neck, including the carotid arteries is presented in the axial- (left), sagittal- (upper right) as well as the coronal (lower right) planes. The image was created using MIM, MIM Software version 6.9.3 (MIM Software Inc. Cleveland, OH, USA, www.mimsoftware.com.

The patients were investigated in a simultaneous whole-body PET/MRI scanner (Signa PET/MR, GE Healthcare, Waukesha, WI, USA) (Fig. 1). The PET unit was equipped with digital detectors, combined with at 3T MR unit. After a minimum 6-h fast, all patients received an intravenous injection of 3 MBq ^18^F-FDG per kg bodyweight. Blood glucose levels were measured prior to the ^18^F-FDG injection and were confirmed to be below 10 mmol/l in all patients. After the ^18^F-FDG injection, the patients spent 90 min in a quiet and warm room to allow for circulation and tissue uptake of the metabolic tracer. Subsequently, a 5 min static PET scan was acquired covering the area from the aortic arch to the lower pole of the kidneys. Then, a 25 min static PET/MRI acquisition was performed over the carotid arteries. The T1-weighted (T1W) sequence, used for segmentations, had slice thickness 2.5 mm and in-plane resolution 0.31 × 0.31 mm. Slice thickness from the PET was 2.78 mm. Thorax images were reconstructed using time-of-flight ordered subset expectation maximization including resolution recovery using 2 iterations, 28 subsets and a 3 mm gaussian post-filter, with a 192 × 192 matrix and a reconstructed field of view (FOV) of 60 cm resulting in a pixel size of 3.1 mm. Images over the neck were reconstructed using time-of-flight block-sequential regularized expectation maximization (BSREM) including resolution recovery (Q.Clear, GE Healthcare, Waukesha, WI, USA) applying a beta value of 50, with a 256 × 256 matrix and a 30 cm FOV resulting in a pixel size of 1.2 mm. The effective spatial resolution in the thorax images was circa 5 mm, whereas it was about 3 mm in the neck images.

### Vessel wall segmentation and analysis of plaque composition

**Figure 2:**
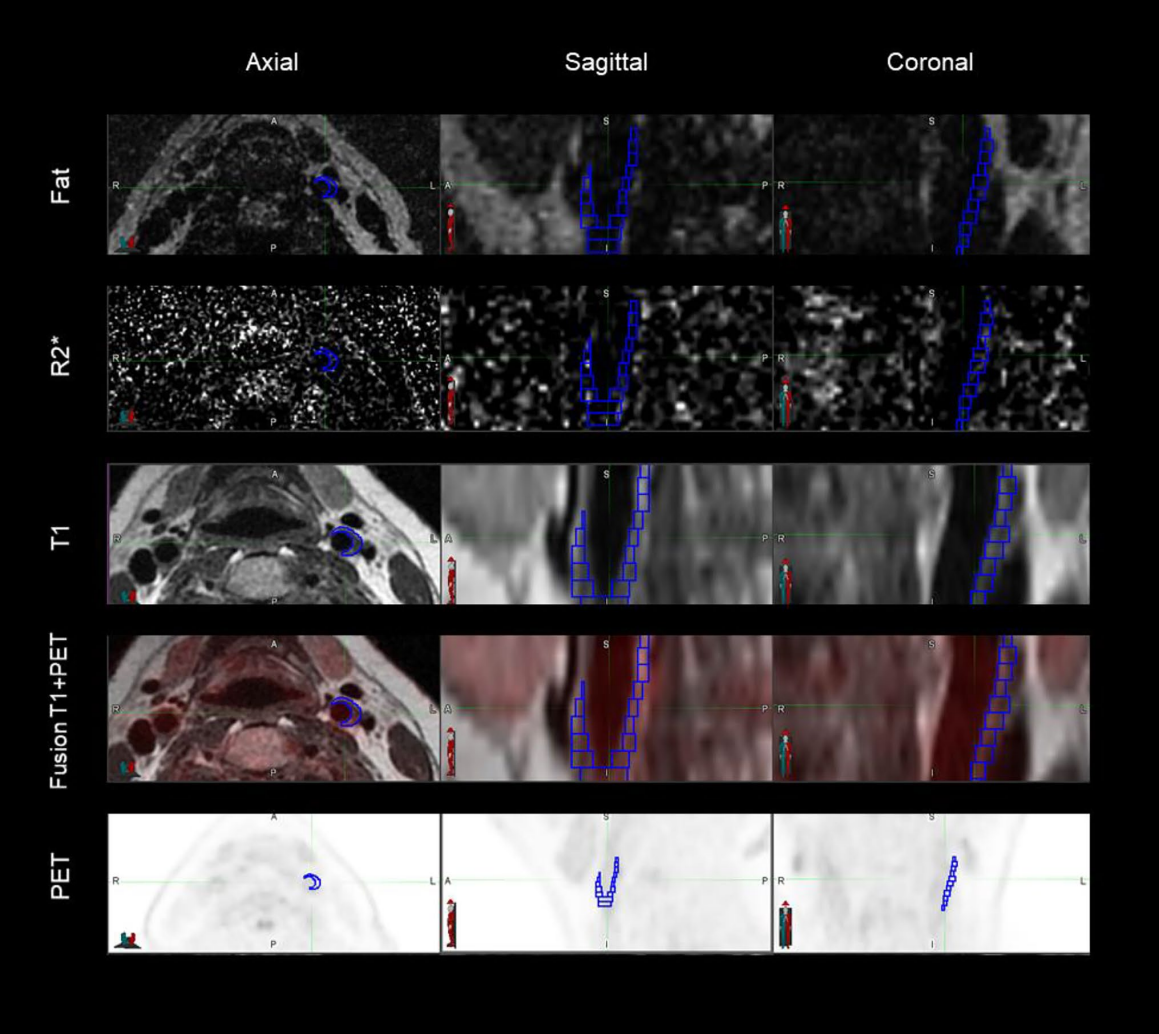
The figure illustrates the principal behind the segmentation and fusion methodology applied in the current study. Segmented carotid plaque is shown in blue, and ^18^F-FDG uptake is shown in red in the corresponding fusion image. The segmented blue volume represents the part of the vessel wall that is thickened, therefore containing the plaque. The plaque visualized in this figure stretches along the lateral part of the carotid wall, explaining why not the circumferent vessel is segmented. Axial, coronal and sagittal planes are automatically aligned for PET, fat, R2* and T1W images. Fat, R2* and T1W images were obtained on a stand-alone scanner using a quantitative MRI protocol. The PET/MRI images were acquired at a national imaging facility. The image was created using MIM, MIM Software version 6.9.3 (MIM Software Inc. Cleveland, OH, USA, www.mimsoftware.com).

Manual segmentation of the carotid artery (Fig. 2) was performed by an experienced reader in vascular MRI (EG) using ITK-SNAP^9^ software and the T1W images from the 3T stand-alone MRI scanner. Segmentations were unilateral. Carotid plaques were delineated in the region of the carotid bifurcation and the internal carotid artery. Plaque was defined as a luminal protrusion of the wall ≥ 1.5 mm in radial thickness. Sampling of FF and R2*, representing compositional information from the vessel wall was done by first registering the manually generated segmentations to the Dixon data. Registrations were performed using MATLAB’s (The MathWorks, Natick, MA, USA) imregister function with a One-Plus-One Evolutionary optimizer^10^ and the Mattes Mutual Information similarity metric^11^. The geometric transformation was nonreflective, and allowed to consist of translation, rotation, and scaling. Registrations were visually inspected and manually corrected if necessary. Next, the average R2* and FF value at each voxel was calculated as an average of its immediate neighbours that were within the mask. However, only slice average and whole mask average for FF and R2* were used in the results.

PET/MRI images were correspondingly segmented using MIM, MIM Software version 6.9.3 (MIM Software Inc. Cleveland, OH, USA, www.mimsoftware.com). The two T1W sets were registered using a nearest neighbour principle, using the bifurcation as a landmark, the data manually aligned according to the slice thickness. This enabled quantitative analysis of R2* and FF versus ^18^F-FDG uptake.

### Analysis of 18F-FDG-uptake in atherosclerotic plaque

Neck images were reconstructed using BSREM because this method achieves the highest spatial resolution, a prerequisite for quantifying tracer uptake in small structures. The long scan time resulted in the acquisition of a high data volume enabling the use of a very small regularization parameter β (50, compared to 350 recommended by the manufacturer for whole-body ^18^F-FDG scans), further improving spatial resolution. Using phantom measurements, we have previously established that a β value of 50 results in a spatial resolution of approximately 2.8 mm (unpublished data). For the thorax images, used for measurements in the venous blood pool, this was not considered necessary since measurement of blood pool activity does not require this high spatial resolution, and hence the faster regular ordered subset expectation maximization (OSEM) was used.

^18^F-FDG uptake in the plaque was expressed as standardized uptake value (SUV) and target-to-blood pool ratio (TBR) and calculated according to current literature^12,13^. To obtain TBR, the mean standardized uptake value (SUV_mean_) was calculated for each region of interest (ROI) within each plaque slice. Then, the SUV_mean_ for the blood pool was calculated as the average of the SUV acquired from six points in the venous blood pool. Subsequently, the TBR was expressed as the SUV_mean_ from the plaque divided by the SUV_mean_ from the blood pool.

### Statistical analysis

IBM SPSS Statistics for Windows, version 26.0 (Armonk, NY, USA, www.ibm.com) was used for statistical analysis. Continuous variables were summarized as mean ± standard deviation (SD). The strength of the association between FF, R2* and TBR was assessed by calculating the Pearson correlation coefficient. Simple linear regression was performed, and all graphs were created using GraphPad Prism version 9.0.0 for Windows (GraphPad Software, San Diego, California USA, www.graphpad.com).

### Ethics approval

The study was approved by the Swedish Ethical Review Authority (approval number 2017/545-31).

### Consent to participate

Written informed consent was obtained from all study participants.

### Consent for publication

Written informed consent was obtained from all study participants.

## Results

### Cardiovascular risk factors

Table 1 summarizes the clinical baseline data of the study participants and their current medical treatment. The majority were men, all were advanced in age and most had clinical evidence of atherosclerotic disease in multiple vascular beds. All were well treated regarding cardiovascular risk factors, with well controlled cholesterol levels and lipid lowering treatment before study start. One patient was on ezetimibe only, all the others had statin-treatment as part of their medication (Table 1).

Patient 4 experienced two ischemic strokes after 19 and 21 months, respectively. These were the only cardiovascular events during 2-year follow up.

### MRI plaque characteristics

The plaques were heterogenous with respect to their volumes and composition, and variations in anatomy between patients resulted in a wide range of the number of slices that were analyzed per plaque. The mean values for the entire group were as follows: FF 0.17% (± 0.07), R2* 47.6 s^−1^ (± 10.9), TBR 1.49 (± 0.48). At group level there was no correlation between mean FF and mean R2* (Supplementary Fig. S1). Compositional data and volumes are shown in Supplementary Tables S1–S12 on a slice-by-slice basis.

### ^18^F-FDG uptake in plaques

The logistics of scheduling the off-site PET/MRI examination resulted in a delay between qMRI and PET/MRI that was on average 71 days (14–219 days). As shown in Supplementary Table S13, neither patients with a time gap in the lowest tertile (14–22 days) nor patients with a time gap in the highest tertile (99–219 days) presented with significant correlations between TBR and plaque compositional data at sub-group level. Furthermore, as shown in Supplementary Figs. S2 and S3, there was no general tendency towards a higher correlation for those patients who had a short time period between examinations, compared to those who had a longer time elapsed. The SUV_max_, SUV_mean_ and TBR from the plaques are shown in Table 2. As expected, the ^18^F-FDG uptake in plaques was higher than in the venous blood pool. The highest ^18^F-FDG uptake in terms of SUV_max_ was seen in Patient 3. This patient was the only subject not receiving statins, instead being treated with ezetimibe. The highest TBR was seen in patient 5.

**Table 2:** Intraplaque ^18^Fluorodeoxyglucose uptake and target-to-blood pool ratios.

Patient	Plaque			Inferior vena cava	
	SUV_max_	SUV_mean_	TBR	SUV_max_	SUV_mean_
Patient 1	3.55	2.13	1.60	2.8	1.33
Patient 2	3.13	1.99	1.81	2.5	1.10
Patient 3	10.73	4.54	1.54	6.2	2.95
Patient 4	2.30	1.65	1.04	3.7	1.58
Patient 5	4.67	2.32	2.68	3.5	0.87
Patient 6	1.73	1.09	1.17	1.8	0.93
Patient 7	3.06	2.08	1.10	3.2	1.88
Patient 8	2.82	1.93	1.27	2.5	1.52
Patient 9	2.92	1.88	1.12	3.7	1.68
Patient 10	3.11	1.86	1.24	3.0	1.50
Patient 11	3.32	1.85	2.02	3.7	0.92
Patient 12	3.50	1.96	1.32	4.5	1.48

The plaque TBR expresses local ^18^Fluorodeoxyglucose uptake in relation to SUV_mean_ in the rest of the blood pool. TBR is calculated according to the method presented by Rudd 2007 and Metha 2012. First the mean SUV value for the entire plaque was calculated based on assessment of SUV in each region of interest (ROI) in each plaque slice. Then the mean SUV for the blood pool was calculated based on SUV from six points in the venous blood pool. Finally, TBR was calculated as follows: TBR = (plaque SUV_mean_)/(venous blood pool SUV_mean_).

S*U*V standard uptake value, *TBR* target-to-blood pool ratio.

### Correlation between plaque compositional data and ^18^F-FDG uptake

**Figure 3:**
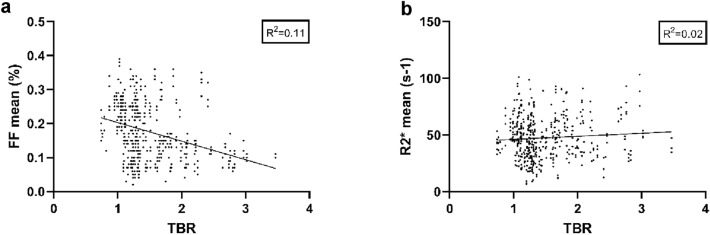
The figures show correlation plots, with plaque composition versus target-to-blood pool ratio. The plots illustrate plaque correlations for all 12 patients, based on the corresponding mean values in each segmented volume. (**a**) Correlations between mean FF and plaque TBR. (**b**) Correlations between R2* and plaque TBR. Simple linear regression was performed using GraphPad Prism version 9.0.0 for Windows, GraphPad Software, San Diego, California USA, www.graphpad.com. *FF* fat fraction, *TBR* target-to-blood pool ratio.

**Figure 4:**
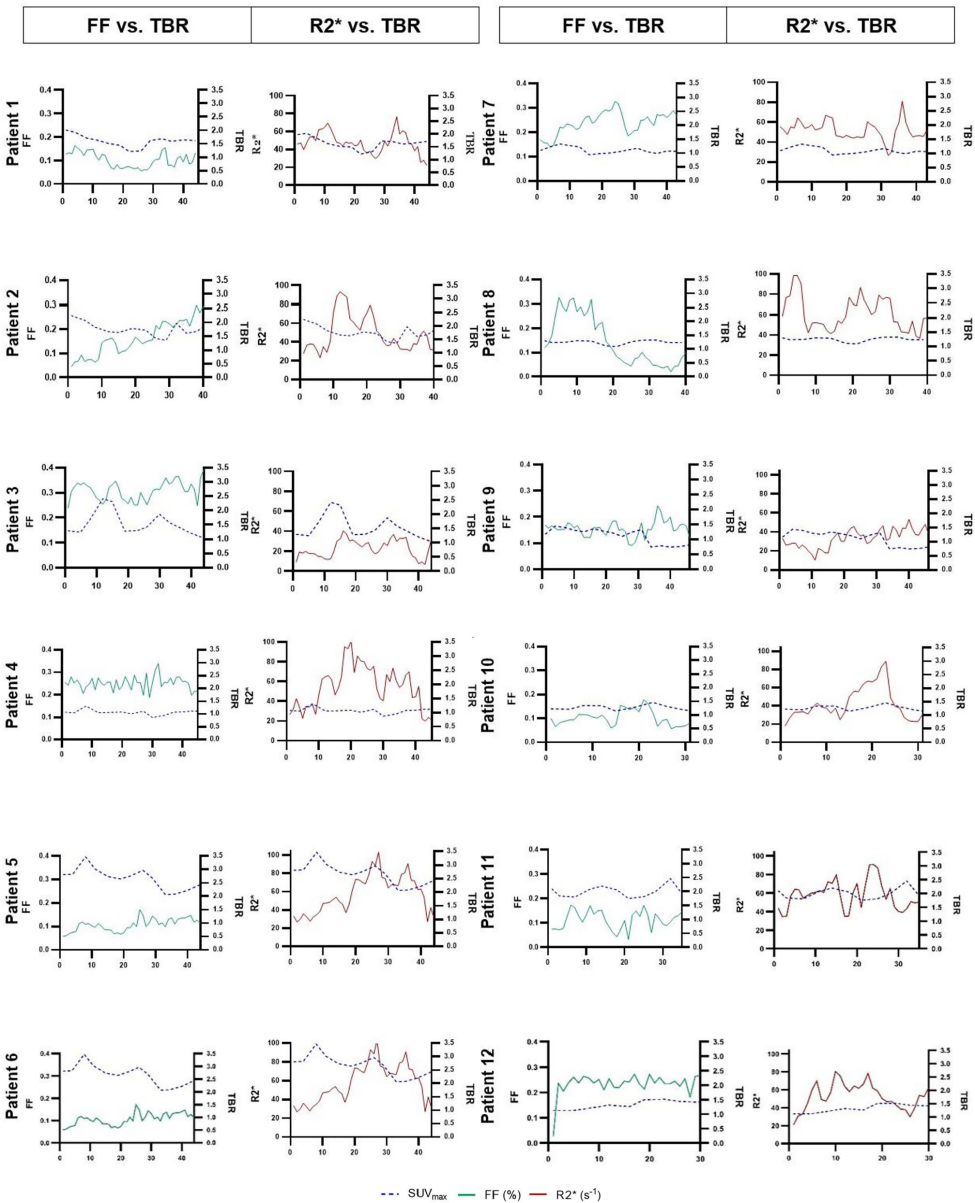
Slice by slice comparison throughout the plaques of target-to-blood pool ratio versus fat fraction and intraplaque hemorrhage. The left panels in the columns show FF and TBR per MRI slice, the right panels R2* and TBR per MRI slice. TBR values are shown on the right y-axis in the figures. The slice numbers are shown on the x- axis, slice numbers differ, as plaque length varies between the patients. The comparison of the 12 patients shows a large heterogeneity in both FF and R2* dispersion and no significant correlation between FF and TBR and respectively R2* and TBR. All graphs were created using GraphPad Prism version 9.0.0 for Windows, GraphPad Software, San Diego, California USA, www.graphpad.com. *FF* fat fraction, *TBR* target-to-blood pool ratio.

At group level there was no significant correlation between TBR and FF_mean_ (− 0.406, *p* 0.19) nor for TBR and R2*_mean_ (0.259, *p* 0.42). Figure 3 shows the correlation plots for the compositional data and TBR based on plaque data from all patients, and Supplementary Fig. S4 shows correlation plots for each individual. Supplementary Fig. S5 presents Pearson correlation coefficients for each patient. None of these coefficients has the threshold of R^2^ 0.5. Thus, also at an individual level there were no strong correlations between plaque compositional data and ^18^F-FDG uptake. Figure 4 shows the mean values for FF and TBR on a slice-by-slice basis along the length of each plaque and the same slice-by-slice presentation is shown for R2* and TBR. These figures illustrate the heterogeneity of plaque composition and ^18^F-FDG uptake. Also, this slice-by-slice analysis showed no statistically significant correlation between lipid rich necrotic core expressed as FF and TBR. Nor was there a significant correlation between intraplaque hemorrhage expressed as R2* and TBR.

## Discussion

Combining qMRI and PET/MRI we show for the first time that it is feasible to interrogate the relationship between lipid rich necrotic core, intraplaque hemorrhage, and inflammation in a quantitative manner. We included 12 patients with advanced but stable carotid atherosclerosis, who were well treated with LDL cholesterol at target levels that were recommended at the time^14^. We conclude that the optimized treatment may explain why these high-risk patients were remarkably stable during clinical follow-up. The plaques were heterogenous with respect to volume and composition. In this pilot study we were unable to find any significant correlations between FF, R2* and ^18^F-FDG uptake, neither at group level, nor on an individual basis.

This is the first time that lipid rich necrotic core and intraplaque hemorrhage have been assessed simultaneously and correlated to ^18^F-FDG uptake using PET/MRI in a quantitative manner. Lipid rich necrotic core as a single measure has previously been studied using PET/CT, demonstrating a significantly higher ^18^F-FDG uptake in carotid and femoral plaques with large lipid cores, compared to plaques with lesser lipid contents^15^. The relationship between lipid rich necrotic core, intraplaque hemorrhage and ^18^F-FDG uptake was investigated in another study, using PET/CT and MRI in stand-alone modalities, showing significantly higher SUV_max_ in plaques with intraplaque hemorrhage as assessed by MRI^16^. Importantly, CT allows for high-resolution imaging and can accurately detect ulceration and calcification. Even so, lipid rich necrotic core and intraplaque hemorrhage cannot be assessed simultaneously on CT because the Hounsfield units of these plaque features are too close to each other to allow for reliable discrimination between the two^17,18^. Previous studies have been less than successful in registering CT images with MRI because the 3D modeling standards based on CT and MRI images are not uniform, especially after image post-processing^19^. However, in the future photon-counting CT scanners potentially might be capable to address this issue and may accurately separate fat and blood.

Regarding MRI, blood-suppressed T1W, T2W, proton-density-weighted fast spin echo, gradient echo and time-of-flight sequences have been used to assess lipid rich necrotic core and intraplaque hemorrhage. This multi-contrast approach relies on extensive post-acquisition assessment of differences in image intensity and therefore it is heavily operator dependent, the sequences are not entirely specific for the plaque features to be studied and the method is not quantitative. In multicontrast carotid plaque imaging, the sternocleidomastoid muscle is used as reference tissue. However, when the signal intensity from the adjacent muscle on fat suppressed T2W images is too low for use as a standard reference, the submandibular and parotid glands have been used^20^. Ideally, the reference tissue should represent a uniform distribution of intensity values, independent of its location within the image volume, but this is rarely the case in the clinic. Finally, when it comes to histological validation of plaque MRI, out-of-plane angulations of the histology sections versus the MRI slices, as well as shrinkage and deformation during tissue processing are routinely neglected^21^. Thus, we developed a qMRI method that directly measures physical properties of plaque, i.e., fat and iron and by registering 3D MRI volumes with 3D histology volumes from the same plaques we were able to demonstrate good agreement (R^2^ = 0.812–0.932) between qMRI measurements and the volumes of lipid rich necrotic core and intraplaque hemorrhage^8^. Thus, we submit that our qMRI method is a reliable readout of the extent of lipid rich necrotic core and intraplaque hemorrhage.

Considering that our patients had advanced carotid atherosclerosis, presenting with both lipid rich necrotic cores and intraplaque hemorrhage, we expected an elevated ^18^F-FDG uptake in the plaques. Instead, we found modest TBR values in most patients. In interpreting these results, several aspects of vascular biology and ^18^F-FDG uptake measurement need to be taken into consideration.

When looking at vascular inflammation in a broader perspective, it is essential to point out that ^18^F-FDG uptake has been shown to be higher in plaque-free segments than in plaques. In a study of 755 middle-aged subjects participating in the Progression of Early Subclinical Atherosclerosis (PESA) clinical trial, vascular inflammation was assessed using ^18^F-FDG PET/MRI^22^. All these patients had known plaques, but carotid plaque inflammation was present in only 15.8% of the patients. Interestingly, increased uptake was located more frequently in plaque-free arterial segments than within plaques. This suggests a complex relationship between ^18^F-FDG uptake and arterial disease.

Adding to this complexity is the observation that ^18^F-FDG accumulation tends to increase during the process of foam cell formation, but decreases to control levels in a later stage of atherosclerosis when differentiation into foam cells is complete^23^. All our patients had advanced atherosclerotic plaques, thus the ratio of fully differentiated foam cells versus undifferentiated foam cells may have been high, possibly contributing to low ^18^F-FDG uptake. Another reason for low ^18^F-FDG uptake could be that plaque foam cells are partially replaced by connective tissue and vascular muscle cells in advanced, stable atherosclerotic disease^24^.

A more general point of concern may be the specificity of the ^18^F-FDG signal for vascular inflammation. ^18^F-FDG accumulation has been attributed to the high glycolytic activity of inflammatory cells, particularly activated macrophages^25^. Nevertheless, glucose transport into the cell is a ubiquitous metabolic process, therefore the continuous glucose uptake from non-macrophage cells limits its specificity. It is undoubtedly so that in atherosclerotic plaques macrophages are the predominant inflammatory cell type, and ^18^F-FDG uptake has been consistently linked to macrophage presence^26,27^. But ^18^F-FDG uptake does not adequately portray the metabolic divergence of macrophages upon activation into pro-inflammatory (M1) or anti-inflammatory (M2) subtypes^28^. Also, data from in-vitro studies indicate hypoxia rather than inflammation as the driver of ^18^F-FDG uptake^29^. These same studies indicate that smooth muscle cells increase their ^18^F-FDG uptake when activated by inflammatory cytokines thus generating a confounding ^18^F-FDG signal, possibly explaining the high uptake in plaque-free regions.

Recently, 18-sodium fluoride (^18^F-NaF) has been used as an alternative PET tracer to ^18^F-FDG, because of several advantages. The ^18^F-NaF molecule is incorporated into areas of calcium deposition by exchanging the hydroxyl ions of hydroxyapatite crystals, forming fluorapatite, and thereby becomes a marker for cardiovascular microcalcification. Microcalcification has been proposed as another indicator of plaque vulnerability^30^. In animal histology studies, the vascular ^18^F-FDG uptake correlates to the extent of calcification^31^. In humans, it is suggested that ^18^F-NaF can be used for the detection of microcalcifications in an early stage in atherosclerosis, even before plaques are detectable by cardiovascular imaging^32^.

In carotid plaques, the general ^18^F-NaF uptake seems to be higher than the uptake of ^18^F-FDG^33^. In fact, ^18^F-NaF seems to have an increased uptake in the carotid bifurcation compared to ^18^F-FDG, which in contrast demonstrates a more diffuse pattern of uptake^34^. One recent study used hybrid PET/MRI for the assessment of ^18^F-NaF uptake in 12 carotid plaques and aimed to correlate the uptake to morphological criteria of plaque vulnerability on MRI^35^. Similar to our results, no such associations were established, however, the methodology for MRI assessment was rudimentarily described and therefore difficult to evaluate.

Currently the numbers of studies using ^18^F-NaF are steadily increasing, challenging ^18^F-FDG as a marker for plaque vulnerability. However, the comparison between ^18^F-FDG and ^18^F-NaF is made difficult by previous variations in methodology, where studies have been using numerous variations in image acquisition methods, analyses and interpretations, resulting in heterogenous data concerning the role of ^18^F-FDG in cardiovascular imaging^36^. There are indications that ^18^F-NaF might be the better choice for diagnosing early changes in atherosclerosis, but, for the current study this advantage would be irrelevant because of the advanced stage of atherosclerosis in our patients. However, in addition to lipid rich necrotic core and intraplaque hemorrhage as markers for plaque vulnerability, calcification is emerging as an additional high-risk feature. In view of the higher specificity of ^18^F-NaF for calcium, this tracer may develop into a useful marker of plaque vulnerability.

The current study was designed as a pilot study and therefore lacked the statistical power to establish significant correlations between ^18^F-FDG and plaque components. The group size was comparable to the number of participants in a majority of other PET/MR studies of carotid plaque^37^, but too small to draw definitive conclusions about the relationship between clinical events and ^18^F-FDG uptake values. However, the clinical events in our patient group still merit consideration. We observed two strokes in Patient 4, who had modest TBR and SUV_max_ values. SUV_max_ was highest in patient 3, who had a relatively low TBR, especially compared to patient 5 who presented the highest TBR. SUV_max_ indicates the most intense voxel activity within a volume of interest, but it may not accurately represent the lesion’s overall activity if the tracer is not homogenously distributed within the plaque. For this reason, TBR is used in vascular imaging as this measure is thought to represent vascular plaque tracer activity more accurately. However, TBR has its own limitations as it is dependent on variable factors that reduce accuracy; for example ^18^F-FDG blood pool activity can fluctuate for biological reasons such as liver- and renal function, and there are also variations depending on the regions selected for the blood pool that serves as denominator in the equation that is used to calculate TBR^38^. The inherent limitations of SUV_max_ and TBR as readouts for ^18^F-FDG uptake together with the cellular and metabolic complexity of atherosclerosis, may account for the lack of agreement between SUV_max_ and TBR and clinical outcomes.

Thus far, it has not been possible to define a universal value for SUV_max_ or TBR that reliably announces plaque inflammation and its extent. Technical variables, such as differences in image acquisition protocols, postfiltering procedures and the number of iterations used, affect the measured SUV and can account for differences in SUV between studies that vary by a factor greater than three^39,40^. The susceptibility of vascular PET to partial-volume effects because of the small size of the vessel wall and the plaques leads to an underestimation of ^18^F-FDG uptake^40^. In our study plaque volume varied considerably between patients and this most likely will have contributed to variations in partial volume effect leading to variations in the degree by which ^18^F-FDG was under-estimated. Yet another level of uncertainty is that the size of the partial volume effect depends on how the images are acquired, reconstructed and analyzed—and here there is no consensus on methodology^41^. To address these uncertainties, we chose an acquisition time of 25 min, rendering a high spatial resolution, and we used the BRSEM reconstruction technique. Our circulation time of 90 min is shorter than the recommended 120 min, but still well beyond the advised minimum of 60 min^41^. Also, our segmentation and delineation of the plaque area is robust and precise, due to our high-resolution MRI imaging.

Taking into consideration the current limitations of ^18^F-FDG imaging for the assessment of plaque inflammation, we submit that our well validated, quantitative imaging techniques, robust registration, and high-resolution reconstruction protocols, provide a framework for analysis of the non-linear and complex interrelationships between plaque composition and plaque inflammation. However, the current study has several limitations. The time delay between stand-alone qMRI and PET/MRI may be considered one of the major concerns. Unfortunately, for logistical reasons this delay was unavoidable. However, patients were clinically stable without any cardiovascular events during the interval between qMRI and PET/MRI, their cardiovascular risk factors were well managed, and their medical regimen remained unchanged during this waiting period. Therefore, we would submit that rapid plaque progression during the interval between the imaging studies was unlikely. This is further supported by our observation that there was no correlation between time delay and the relationship between FF, R2* and TBR (Supplementary Table S13, Supplementary Fig. S2–S3). Another limitation of our study is the fact that 4-point Dixon sequence was not implemented on the PET/MRI scanner. The sequence was developed for qMRI analysis of plaques using a 3 T Philips Ingenia scanner. Our national facility for PET/MRI provides a GE scanner. After thorough analysis we concluded that we could not simply implement the 4-point Dixon sequence on the GE scanner, as this would require a renewed validation of the data processing, preferably with 3D histology. Because PET/MRI was off-site we were unable to overcome the logistical challenges that such a validation would imply. Instead, we relied on high-accuracy registration of qMRI with MRI from PET/MRI, using a slice-by-slice registration with the bifurcation as the primary landmark. The registration is robust as it involves no change in modality (T1W to T1W). We acknowledge that in future studies it is preferable to implement qMRI on the PET/MRI scanner.

## Conclusion

Using well validated quantitative imaging techniques we established feasibility of assessing the relationship between lipid rich necrotic cores and intraplaque haemorrhage measured by qMRI and plaque inflammation as measured by ^18^F-FDG uptake. The ^18^F-FDG uptake in plaques was modest in most of our patients. Here both biological and technical variables must be considered. Among these, questions raised in the literature regarding the specificity of ^18^F-FDG for inflammatory macrophages may indicate the need for more specific tracers for plaque inflammation when embarking on large scale studies that interrogate the complex relationship between lipid rich necrotic core, intraplaque haemorrhage and plaque inflammation.

## Supplementary Information

Supplementary Information 1.

## Data Availability

The datasets generated during and analyzed during the current study are not publicly available due to patient integrity reasons but are available from the corresponding author on reasonable request.
