# Supplementary material for: 18Fluorodeoxyglucose uptake in relation to fat fraction and R2* in atherosclerotic plaques, using PET/MRI: a pilot study
Source: Sci Rep. 2021 Jul 9;11:14217. doi: 10.1038/s41598-021-93605-x (PMC8270927; doi:10.1038/s41598-021-93605-x)
Supplement: Supplementary file 1 — Supplementary Information 1. [file 41598_2021_93605_MOESM1_ESM.pdf]

Supplementary Information

[18]Fluorodeoxyglucose uptake in relation to fat fraction and R2* in atherosclerotic plaques, using PET/MRI: a pilot study.

Elin Good[1,2,3], Miguel Ochoa-Figueroa[2, 4, 5], Magnus Ziegler[1,2], Marcus Ressner[2, 4, 5], Marcel Warntjes[1,2,6], Petter Dyverfeldt[1,2], Mark Lubberink[7], Håkan Ahlström[7,8] and Ebo de Muinck[1,2,3]

[1.] Department of Health, Medicine and Caring Sciences, Linköping University, Linköping, Sweden

[2.] Center for Medical Image Science and Visualization (CMIV), Linköping University, Linköping, Sweden

[3.] Department of Cardiology in Linköping, and Department of Health, Medicine and Caring Sciences, Linköping University, Linköping, Sweden

[4.] Department of Clinical Physiology in Linköping, and Department of Health, Medicine and Caring Sciences, Linköping University, Sweden

[5.] Department of Radiology in Linköping, and Department of Health, Medicine and Caring Sciences, Linköping University, Linköping, Sweden

[6.] SyntheticMR AB, Linköping, Sweden

[7.] Department of Surgical Sciences, Section of Radiology, Uppsala University, Uppsala, Swede

[8.] Antaros Medical AB, Mölndal, Sweden.

Corresponding Author:

Elin Good, Department of Health,
Medicine and Caring Sciences,
Division of Cardiovascular Medicine
Linköping University
SE-581 85 Linköping
Sweden.

Email: elin.good@liu.se

Supplementary Table S1. PET and MRI data from Patient 1

Slice	SUVmax	SUVmean	SUVmedian	SUVmin	FF mean (%)	FF cum (%mm^3)	R2* mean (s^{-1})	R2* cum (mm^3/s)	Voxels (N)	Volume (mm^3)
1	3.55	2.67	2.66	1.72	0.128	7.7	46.0	2762.1	60	15.0
					0.133	7.9	47.4	2797.1	59	14.8
					0.128	7.5	39.8	2347.3	59	14.8
2	3.37	2.56	2.53	1.71	0.164	10.6	47.4	3080.9	65	16.3
					0.152	9.4	53.7	3331.8	62	15.5
					0.141	9.3	54.0	3564.4	66	16.5
					0.120	7.9	49.6	3275.7	66	16.5
3	2.62	2.27	2.14	1.85	0.145	8.4	62.1	3602.0	58	14.5
					0.145	7.4	62.6	3192.3	51	12.8
					0.149	7.7	64.9	3372.5	52	13.0
4	2.7	2.19	2.26	1.77	0.118	6.6	69.3	3883.4	56	14.0
					0.101	5.9	63.4	3676.1	58	14.5
					0.126	7.6	55.5	3327.7	60	15.0
					0.122	11.0	47.8	4304.3	90	22.5
5	2.55	2.01	1.92	1.76	0.092	9.0	47.2	4629.3	98	24.5
					0.069	6.0	44.0	3829.7	87	21.8
					0.063	5.8	48.4	4405.8	91	22.8
					0.083	8.1	46.9	4593.3	98	24.5
6	2.17	1.91	1.9	1.68	0.065	7.8	47.6	5709.7	120	30.0
bifurcation					0.067	8.4	44.3	5580.7	126	31.5
					0.073	9.6	44.7	5902.1	132	33.0
7	1.82	1.61	1.66	1.39	0.074	6.5	50.5	4447.5	88	22.0
					0.068	6.4	39.5	3716.9	94	23.5
					0.065	6.5	35.8	3581.8	100	25.0
					0.069	7.1	38.1	3883.1	102	25.5
8	2.09	1.64	1.66	1.21	0.055	5.6	32.7	3300.2	101	25.3
					0.061	5.9	29.8	2923.1	98	24.5
					0.060	5.6	33.1	3076.6	93	23.3
9	2.54	2.15	2.16	1.92	0.072	6.4	39.1	3438.1	88	22.0
					0.096	6.3	49.0	3186.9	65	16.3
					0.105	6.7	43.7	2799.1	64	16.0
					0.105	6.0	50.4	2874.4	57	14.3
10	2.99	2.24	2.18	1.76	0.146	7.7	62.8	3329.7	53	13.3
					0.155	7.9	76.7	3909.9	51	12.8
					0.078	4.3	57.1	3139.6	55	13.8
11	2.63	2.11	2.21	1.19	0.081	4.5	60.9	3411.6	56	14.0
					0.071	3.8	58.9	3179.9	54	13.5
					0.110	5.5	47.1	2354.6	50	12.5
					0.118	5.1	46.0	1979.6	43	10.8
12	2.59	2.19	2.3	1.21	0.094	3.5	38.3	1415.6	37	9.3
					0.127	5.1	43.2	1729.3	40	10.0
					0.085	3.6	25.4	1065.1	42	10.5
					0.091	3.3	28.0	1007.6	36	9.0
13	2.66	2.15	2.29	1.33	0.131	8.8	22.2	1488.3	67	16.8

Supplementary Table S2. PET and MRI data from Patient 2

Slice	SUVmax	SUVmean	SUVmedian	SUVmin	FF mean (%)	FF cum (%mm^3)	R2* mean (s^{-1})	R2* cum (mm^3/s)	Voxels (N)	Volume (mm^3)
1	3.13	2.47	2.48	1.87	0.045	3.7	27.6	2261.4	82	20.5
					0.065	5.5	36.8	3093.5	84	21.0
					0.068	5.7	38.0	3191.3	84	21.0
					0.094	8.2	37.6	3269.0	87	21.8
2	2.71	2.28	2.23	1.79	0.066	4.9	31.5	2331.5	74	18.5
					0.075	5.7	23.3	1772.3	76	19.0
					0.075	5.3	35.2	2465.2	70	17.5
3	2.96	2.01	1.99	1.47	0.064	4.1	29.0	1855.6	64	16.0
					0.075	4.4	40.4	2384.4	59	14.8
					0.144	7.9	72.2	3973.6	55	13.8
					0.155	8.2	88.1	4669.1	53	13.3
4	2.65	1.83	1.79	1.33	0.159	8.5	93.1	4934.5	53	13.3
					0.164	11.0	90.1	6038.6	67	16.8
					0.145	9.5	87.6	5780.3	66	16.5
5	2.36	1.8	1.82	1.36	0.099	6.4	64.4	4122.7	64	16.0
					0.109	7.4	61.3	4168.2	68	17.0
					0.112	8.2	56.6	4135.0	73	18.3
					0.125	9.7	52.7	4110.6	78	19.5
6	2.36	1.96	1.98	1.29	0.145	11.3	64.9	5064.1	78	19.5
					0.168	14.4	70.8	6090.1	86	21.5
					0.155	14.5	79.6	7399.9	93	23.3
					0.140	12.6	67.7	6095.6	90	22.5
7	2.31	1.89	1.9	1.48	0.148	12.9	51.9	4516.2	87	21.8
bifurcation					0.153	13.6	48.0	4271.3	89	22.3
					0.156	13.2	39.9	3388.1	85	21.3
8	2.09	1.56	1.49	1.29	0.216	10.8	36.2	1808.4	50	12.5
					0.200	10.0	36.7	1836.6	50	12.5
					0.233	12.8	44.0	2419.6	55	13.8
9	2.35	1.49	1.38	1.17	0.232	13.5	39.3	2279.9	58	14.5
					0.222	12.0	32.6	1759.6	54	13.5
					0.210	10.9	32.2	1672.3	52	13.0
10	2.45	2.17	2.27	1.63	0.221	12.4	32.3	1808.4	56	14.0
					0.237	11.4	30.4	1460.4	48	12.0
					0.236	7.3	38.3	1186.1	31	7.8
11	2.77	1.77	1.62	0.97	0.242	7.3	37.6	1127.7	30	7.5
					0.213	8.3	47.3	1844.9	39	9.8
					0.254	10.4	51.6	2116.7	41	10.3
12	2.47	1.84	1.79	1.32	0.300	11.4	47.1	1789.8	38	9.5
					0.268	8.6	32.0	1024.4	32	8.0
					0.297	9.2	31.4	972.4	31	7.7
13	2.57	2.11	2.2	1.35	0.271	7.0	33.4	868.4	26	6.5
					0.269	6.7	57.7	1442.2	25	6.3
					0.259	6.5	57.9	1446.3	25	6.3
14	3.13	2.47	2.48	1.87	0.045	3.7	27.6	2261.4	82	20.5
					0.065	5.5	36.8	3093.5	84	21.0
					0.068	5.7	38.0	3191.3	84	21.0

Supplementary Table S3. PET and MRI data from Patient 3

Slice	SUVmax	SUVmean	SUVmedian	SUVmin	FF mean (%)	FF cum (%mm^3)	R2* mean (s^{-1})	R2* cum (mm^3/s)	Voxels (N)	Volume (mm^3)
1	4.95	3.81	3.81	2.5	0.238	10.5	9.1	400.8	44	11.0
					0.304	10.3	19.1	650.4	34	8.5
					0.323	11.0	18.0	611.1	34	8.5
					0.339	11.5	19.8	673.2	34	8.5
2	5.23	3.67	3.89	1.63	0.331	17.2	17.8	923.5	52	13.0
					0.339	20.3	17.7	1063.7	60	15.0
					0.328	20.0	17.2	1051.2	61	15.3
3	6.97	5	4.93	2.36	0.320	18.6	14.6	844.4	58	14.5
					0.302	19.3	14.7	940.4	64	16.0
					0.278	19.2	12.4	853.1	69	17.2
					0.269	19.1	11.7	829.1	71	17.8
4	10.34	7.11	7.29	2.88	0.253	19.4	12.4	954.1	77	19.3
					0.271	18.5	19.9	1352.7	68	17.0
					0.322	21.0	30.1	1955.8	65	16.3
5	10.73	6.82	6.41	2.97	0.331	22.2	32.1	2152.3	67	16.8
					0.346	27.4	40.4	3192.9	79	19.8
					0.321	25.6	37.4	2995.5	80	20.0
					0.277	24.1	30.3	2631.8	87	21.8
6	5.51	3.77	3.77	2.23	0.269	23.1	31.2	2682.6	86	21.5
					0.256	20.7	29.0	2351.1	81	20.3
					0.282	24.0	26.4	2247.0	85	21.3
					0.250	20.7	24.2	2006.0	83	20.8
7	5.12	3.82	3.64	2.81	0.250	19.5	27.3	2132.1	78	19.5
bifurcation					0.301	26.5	28.7	2528.3	88	22.0
					0.274	23.5	21.2	1821.0	86	21.5
8	8.79	4.21	4.2	2.81	0.251	20.6	17.4	1429.9	82	20.5
					0.284	23.9	18.6	1559.9	84	21.0
					0.309	25.6	23.1	1921.0	83	20.8
					0.314	23.5	28.2	2116.0	75	18.8
9	10.12	5.52	5.24	3.06	0.312	26.2	25.5	2143.3	84	21.0
					0.336	27.2	33.0	2669.1	81	20.3
					0.359	30.9	36.9	3177.4	86	21.5
10	8.31	4.67	4.05	2.75	0.334	30.4	29.6	2696.5	91	22.8
					0.341	23.2	33.1	2248.6	68	17.0
					0.363	21.1	32.7	1897.8	58	14.5
					0.365	20.4	33.8	1892.5	56	14.0
11	4.8	4.02	3.95	2.91	0.333	34.9	21.5	2258.9	105	26.2
					0.305	32.1	15.0	1573.1	105	26.3
					0.304	31.3	13.4	1383.6	103	25.8
12	4.55	3.54	3.47	2.55	0.334	12.4	7.4	273.5	37	9.2
					0.313	10.3	9.3	306.8	33	8.3
					0.247	6.9	6.6	185.5	28	7.0
					0.362	12.3	19.2	653.9	34	8.5
13	3.68	3.05	3.05	2.18	0.393	14.9	32.0	1214.9	38	9.5
					0.377	11.3	23.5	705.4	30	7.5
					0.367	17.6	27.4	1313.8	48	12.0

Supplementary Table S4. PET and MRI data from Patient 4

Slice	SUVmax	SUVmean	SUVmedian	SUVmin	FF mean (%)	FF cum (%mm^3)	R2* mean (s^{-1})	R2* cum (mm^3/s)	Voxels (N)	Volume (mm^3)
1	2.07	1.7	1.67	1.27	0.255	8.9	26.7	933.8	35	8.8
					0.243	8.5	31.6	1104.9	35	8.7
					0.280	9.2	42.7	1407.7	33	8.2
2	2.07	1.66	1.63	1.22	0.252	10.8	33.5	1441.0	43	10.8
					0.266	11.7	22.6	995.0	44	11.0
					0.246	9.1	34.9	1290.6	37	9.3
					0.206	6.8	34.2	1128.8	33	8.3
3	2.07	1.59	1.58	1.06	0.236	9.4	36.6	1463.3	40	10.0
					0.247	8.4	26.8	912.4	34	8.5
					0.274	13.7	46.7	2334.3	50	12.5
					0.222	11.3	62.4	3180.4	51	12.8
4	2.25	1.67	1.64	1.35	0.276	16.0	64.9	3764.5	58	14.5
bifurcation					0.252	14.9	65.9	3889.9	59	14.8
					0.243	13.6	61.7	3457.5	56	14.0
5	2.02	1.68	1.67	1.36	0.257	16.2	46.4	2923.3	63	15.8
					0.257	16.2	53.1	3345.4	63	15.8
					0.208	13.1	64.3	4049.1	63	15.8
					0.267	14.7	95.3	5242.0	55	13.8
					0.223	13.2	93.2	5499.7	59	14.8
6	2.15	1.71	1.71	1.29	0.239	9.5	100.9	4036.1	40	10.0
					0.282	11.3	68.9	2755.9	40	10.0
					0.255	9.5	85.9	3179.7	37	9.3
7	1.86	1.59	1.61	1.38	0.221	9.5	80.7	3471.7	43	10.8
					0.288	13.0	80.1	3603.5	45	11.3
					0.254	9.7	73.3	2786.1	38	9.5
					0.255	10.5	71.4	2927.6	41	10.3
8	2.07	1.76	1.74	1.36	0.197	7.1	75.6	2722.9	36	9.0
					0.298	4.5	52.1	780.8	15	3.8
					0.187	4.3	42.6	980.4	23	5.8
9	1.61	1.37	1.38	1.07	0.253	6.6	40.6	1055.1	26	6.5
					0.312	12.2	67.0	2614.1	39	9.8
					0.340	13.3	59.7	2328.9	39	9.8
					0.236	5.9	73.7	1842.5	25	6.2
10	1.75	1.5	1.47	1.26	0.257	8.0	60.9	1887.5	31	7.7
					0.253	8.1	53.9	1723.9	32	8.0
					0.275	11.6	57.0	2396.0	42	10.5
11	2.06	1.7	1.72	1.16	0.246	11.8	66.7	3201.9	48	12.0
					0.279	12.8	69.9	3216.0	46	11.5
					0.280	10.6	43.6	1658.6	38	9.5
12	2.2	1.71	1.66	1.16	0.259	10.4	44.5	1781.0	40	10.0
					0.261	9.1	55.1	1927.9	35	8.8
					0.251	5.5	22.3	491.4	22	5.5
13	2.3	1.77	1.7	1.38	0.198	4.9	19.7	492.6	25	6.2
					0.217	5.6	23.7	617.2	26	6.5
					0.215	5.6	20.7	538.4	26	6.5

Supplementary Table S5. PET and MRI data from Patient 5

Slice	SUVmax	SUVmean	SUVmedian	SUVmin	FF mean (%)	FF cum (%mm^3)	R2* mean (s^{-1})	R2* cum (mm^3/s)	Voxels (N)	Volume (mm^3)
1	3.03	2.44	2.41	1.65	0.058	4.8	33.2	2787.2	84	21.0
					0.061	5.0	26.8	2198.7	82	20.5
					0.070	5.7	30.0	2456.4	82	20.5
2	4.66	2.46	2.36	1.08	0.075	6.1	36.0	2954.0	82	20.5
					0.078	6.9	32.4	2883.3	89	22.3
					0.112	11.2	27.7	2765.3	100	25.0
					0.118	11.4	32.4	3113.1	96	24.0
3	4.67	3.02	2.64	1.83	0.109	10.4	34.9	3310.9	95	23.8
					0.112	14.6	38.3	4983.9	130	32.5
					0.102	13.2	47.1	6128.7	130	32.5
4	4.67	2.63	2.21	1.69	0.096	14.2	47.6	7051.4	148	37.0
					0.112	16.7	48.6	7291.5	150	37.5
					0.101	14.7	51.4	7505.7	146	36.5
					0.086	8.2	53.9	5123.8	95	23.8
5	3.19	2.38	2.43	1.53	0.086	8.4	48.8	4778.7	98	24.5
					0.084	7.6	46.3	4163.8	90	22.5
					0.068	6.2	37.0	3405.7	92	23.0
					0.073	6.3	46.7	4064.1	87	21.8
6	3.19	2.3	2.26	1.55	0.067	7.0	61.9	6440.9	104	26.0
bifurcation					0.073	9.2	73.5	9258.3	126	31.5
					0.095	12.0	73.0	9196.2	126	31.5
7	3.67	2.4	2.15	1.5	0.094	17.0	70.3	12726.1	181	45.3
					0.115	21.2	68.6	12615.5	184	46.0
					0.099	15.8	78.5	12564.8	160	40.0
					0.171	13.0	93.1	7077.6	76	19.0
8	3.69	2.58	2.5	1.67	0.149	10.0	88.5	5930.1	67	16.8
					0.099	9.6	103.4	10028.1	97	24.3
					0.115	13.0	75.6	8545.5	113	28.3
9	2.79	2.33	2.46	1.73	0.144	16.8	74.4	8706.4	117	29.3
					0.132	21.2	64.1	10317.9	161	40.2
					0.108	18.5	67.7	11579.1	171	42.8
					0.115	18.6	67.4	10924.5	162	40.5
10	2.31	1.8	1.78	1.08	0.098	14.4	66.5	9706.5	146	36.5
					0.130	18.9	79.0	11449.2	145	36.3
					0.137	10.6	80.4	6193.5	77	19.3
11	2.07	1.81	1.85	1.29	0.110	7.7	90.9	6361.4	70	17.5
					0.131	9.2	72.4	5071.3	70	17.5
					0.135	14.0	72.0	7485.5	104	26.0
					0.135	14.8	62.4	6859.6	110	27.5
12	2.36	1.93	2.02	1.14	0.141	13.8	62.8	6150.5	98	24.5
					0.147	8.2	53.8	3010.5	56	14.0
					0.118	6.2	27.0	1429.4	53	13.3
					0.127	6.6	42.8	2227.9	52	13.0
13	2.67	2.12	2.09	1.65	0.113	6.6	30.6	1773.1	58	14.5

Supplementary Table S6. PET and MRI data from Patient 6

Slice	SUVmax	SUVmean	SUVmedian	SUVmin	FF mean (%)	FF cum (%mm^3)	R2* mean (s^{-1})	R2* cum (mm^3/s)	Voxels (N)	Volume (mm^3)
1	1.54	1.23	1.28	0.95	0.121	4.1	59.3	2015.5	34	8.5
					0.136	5.7	49.9	2096.2	42	10.5
					0.132	5.9	35.4	1594.9	45	11.3
					0.145	6.8	25.6	1205.1	47	11.8
2	1.60	1.17	1.16	0.85	0.104	3.5	31.3	1063.2	34	8.5
					0.093	3.2	29.9	1016.0	34	8.5
					0.062	2.0	34.6	1140.2	33	8.3
3	1.73	1.08	1.05	0.82	0.053	1.9	34.7	1250.7	36	9.0
					0.067	2.4	51.4	1852.0	36	9.0
					0.100	4.4	71.8	3160.5	44	11.0
					0.145	12.2	61.4	5159.4	84	21.0
4	1.12	0.98	0.99	0.81	0.202	17.4	52.9	4548.4	86	21.5
					0.261	16.9	50.3	3269.6	65	16.3
					0.261	20.1	58.5	4502.6	77	19.3
					0.205	14.8	58.4	4203.7	72	18.0
5	1.10	0.92	0.91	0.71	0.194	12.8	68.3	4510.7	66	16.5
bifurcation					0.167	10.7	71.2	4559.3	64	16.0
					0.177	9.0	60.7	3094.5	51	12.8
6	1.10	0.92	0.94	0.74	0.252	14.4	48.0	2737.3	57	14.3
					0.178	10.2	52.5	2989.8	57	14.3
					0.177	11.1	61.6	3883.0	63	15.8
					0.211	15.4	56.0	4087.2	73	18.3
7	1.11	0.95	0.95	0.80	0.224	16.3	57.0	4163.8	73	18.3
					0.189	12.5	45.2	2981.6	66	16.5
					0.216	14.7	50.6	3440.1	68	17.0
8	1.19	1.03	1.03	0.93	0.243	16.7	48.2	3324.3	69	17.3
					0.225	15.9	38.5	2730.2	71	17.8
					0.220	14.8	31.1	2083.7	67	16.8
					0.163	10.8	47.7	3150.6	66	16.5
9	1.51	1.30	1.29	1.00	0.131	7.6	38.7	2246.9	58	14.5
					0.204	9.6	31.0	1455.2	47	11.8
					0.199	7.4	26.6	984.5	37	9.3
10	1.70	1.22	1.18	0.94	0.233	9.1	24.4	951.3	39	9.8
					0.244	5.4	39.2	863.1	22	5.5
					0.149	6.4	41.1	1765.4	43	10.8
					0.146	6.5	36.5	1642.7	45	11.3
11	1.26	1.15	1.16	0.95	0.128	5.4	43.7	1835.8	42	10.5
					0.125	5.6	47.8	2153.1	45	11.3
					0.090	3.7	44.3	1815.9	41	10.3
					0.076	2.6	20.8	708.4	34	8.5
12	1.22	1.09	1.08	1.02	0.076	2.5	22.3	737.3	33	8.2
					0.075	1.9	25.6	639.0	25	6.3
					0.123	3.4	29.6	828.4	28	7.0

Supplementary Table S7. PET and MRI data from Patient 7

Slice	SUVmax	SUVmean	SUVmedian	SUVmin	FF mean (%)	FF cum (%mm^3)	R2* mean (s^{-1})	R2* cum (mm^3/s)	Voxels (N)	Volume (mm^3)
1	2.7	2.07	2.11	1.28	0.169	13.3	55.4	4376.6	79	19.8
					0.157	14.4	52.3	4808.0	92	23.0
					0.156	14.8	47.6	4519.5	95	23.8
2	2.93	2.31	2.3	1.7	0.141	12.7	55.4	4986.3	90	22.5
					0.146	12.2	54.2	4498.4	83	20.8
					0.181	15.4	64.3	5464.5	85	21.3
3	3.06	2.5	2.52	1.66	0.221	19.0	59.8	5139.3	86	21.5
					0.218	27.4	54.0	6803.2	126	31.5
					0.233	30.8	54.7	7217.9	132	33.0
4	3.06	2.39	2.27	1.66	0.232	29.2	57.8	7284.2	126	31.5
					0.225	26.7	52.4	6234.7	119	29.8
					0.212	25.0	53.1	6268.4	118	29.5
5	2.82	2.31	2.25	1.87	0.218	29.4	54.8	7394.6	135	33.8
					0.244	32.2	67.1	8857.7	132	33.0
					0.262	32.5	65.4	8111.2	124	31.0
6	2.21	1.78	1.81	1.37	0.267	23.5	64.2	5646.8	88	22.0
bifurcation					0.237	21.3	46.5	4182.7	90	22.5
					0.246	26.5	45.7	4930.7	108	27.0
7	2.18	1.85	1.84	1.22	0.267	36.8	44.7	6169.9	138	34.5
					0.282	39.2	46.9	6516.8	139	34.8
					0.293	38.7	45.0	5943.0	132	33.0
8	2.13	1.89	1.9	1.64	0.288	38.0	44.1	5823.9	132	33.0
					0.294	36.5	45.2	5604.0	124	31.0
					0.327	33.7	44.6	4595.2	103	25.8
9	2.14	1.97	1.96	1.78	0.318	33.4	45.0	4725.8	105	26.3
					0.280	26.3	60.5	5683.4	94	23.5
					0.225	19.3	57.3	4923.6	86	21.5
10	2.57	2.1	2.06	1.78	0.185	15.4	55.5	4607.0	83	20.8
					0.195	14.1	51.1	3680.2	72	18.0
					0.207	12.4	46.3	2778.5	60	15.0
11	2.62	2.21	2.21	1.98	0.208	12.5	36.5	2192.5	60	15.0
					0.249	14.4	26.5	1536.8	58	14.5
					0.251	13.8	30.5	1676.9	55	13.8
12	2.2	1.97	1.94	1.8	0.264	14.8	51.1	2864.0	56	14.0
					0.243	13.9	59.3	3380.8	57	14.3
					0.228	13.0	81.1	4620.6	57	14.3
13	2.2	1.86	1.88	1.6	0.272	18.5	59.9	4073.7	68	17.0
					0.269	18.8	50.6	3541.7	70	17.5
					0.272	18.5	45.3	3078.9	68	17.0
14	2.66	2.03	1.99	1.66	0.261	9.7	46.4	1718.6	37	9.2
					0.274	11.0	46.5	1858.6	40	10.0
					0.290	11.3	45.5	1774.5	39	9.8
15	2.78	2	1.89	1.51	0.269	12.7	52.2	2454.6	47	11.8
					0.222	12.2	42.7	2347.4	55	13.8
					0.195	9.3	40.1	1927.0	48	12.0

Supplementary Table S8. PET and MRI data from Patient 8

Slice	SUVmax	SUVmean	SUVmedian	SUVmin	FF mean (%)	FF cum (%mm^3)	R2* mean (s^{-1})	R2* cum (mm^3/s)	Voxels (N)	Volume (mm^3)
1	2.24	1.98	2.01	1.37	0.120	4.8	58.9	2356.7	40	10.0
					0.135	7.2	76.7	4064.6	53	13.3
2	2.23	1.89	1.88	1.54	0.170	8.7	78.1	3984.3	51	12.8
					0.252	6.8	98.5	2659.7	27	6.8
					0.325	9.1	98.6	2761.5	28	7.0
					0.291	9.3	90.5	2895.6	32	8.0
3	2.39	1.91	1.94	1.38	0.270	11.9	54.0	2377.3	44	11.0
					0.314	13.8	42.3	1859.6	44	11.0
					0.324	13.0	52.0	2078.5	40	10.0
4	2.23	1.98	1.97	1.53	0.269	13.7	52.4	2673.8	51	12.8
					0.290	13.3	51.6	2375.1	46	11.5
					0.252	11.8	50.7	2381.5	47	11.8
					0.271	12.2	44.2	1990.5	45	11.3
5	2.16	1.96	1.94	1.69	0.318	14.9	41.6	1957.2	47	11.8
					0.194	10.8	43.9	2459.4	56	14.0
					0.217	13.0	54.3	3259.2	60	15.0
					0.225	11.9	51.6	2735.4	53	13.3
6	2.29	1.70	1.68	0.99	0.147	10.3	58.0	4062.6	70	17.5
bifurcation					0.129	9.1	76.8	5452.4	71	17.8
					0.107	6.7	70.3	4359.9	62	15.5
7	2.28	1.66	1.62	1.43	0.083	5.0	69.1	4148.0	60	15.0
					0.073	4.5	86.6	5370.8	62	15.5
					0.059	5.5	73.7	6931.9	94	23.5
					0.052	5.5	68.1	7221.6	106	26.5
8	3.10	1.93	1.90	1.29	0.044	4.3	60.4	5919.3	98	24.5
					0.075	6.4	63.2	5373.0	85	21.3
					0.080	7.0	79.1	6881.5	87	21.8
9	2.84	2.01	1.98	1.39	0.101	9.7	74.9	7186.4	96	24.0
					0.081	8.5	76.8	8058.8	105	26.3
					0.068	6.8	75.8	7501.9	99	24.8
					0.048	3.4	53.1	3772.9	71	17.8
10	2.73	2.02	2.02	1.56	0.047	3.2	52.9	3543.4	67	16.8
					0.040	2.6	43.2	2805.1	65	16.3
					0.036	2.6	42.8	3082.0	72	18.0
11	2.31	1.89	1.77	1.57	0.044	3.0	41.9	2894.4	69	17.3
					0.020	0.6	53.8	1613.9	30	7.5
					0.042	1.4	39.0	1285.9	33	8.3
					0.045	1.7	36.8	1359.9	37	9.2
12	2.19	1.89	1.85	1.52	0.079	3.5	56.6	2547.4	45	11.3
					0.097	4.4	56.8	2555.6	45	11.3
					0.122	5.1	58.3	2450.1	42	10.5

Supplementary Table S9. PET and MRI data from Patient 9

Slice	SUVmax	SUVmean	SUVmedian	SUVmin	FF mean (%)	FF cum (%mm^3)	R2* mean (s^{-1})	R2* cum (mm^3/s)	Voxels (N)	Volume (mm^3)
1	2.73	1.96	2.01	1.25	0.168	13.2	33.1	2612.0	79	19.8
					0.157	10.5	26.0	1739.4	67	16.8
					0.166	10.4	26.8	1689.7	63	15.8
2	2.74	2.42	2.44	1.89	0.157	8.8	28.2	1576.7	56	14.0
					0.151	8.1	27.9	1505.8	54	13.5
					0.152	9.8	22.7	1452.5	64	16.0
3	2.89	2.36	2.37	1.48	0.152	9.3	26.2	1598.4	61	15.3
					0.177	11.1	23.8	1498.9	63	15.8
					0.172	12.1	19.8	1383.9	70	17.5
4	2.76	2.19	2.21	1.46	0.158	9.1	16.4	952.5	58	14.5
					0.167	7.7	10.4	476.9	46	11.5
					0.146	7.7	22.7	1201.7	53	13.3
5	2.92	2.11	2.09	1.46	0.121	8.1	21.0	1410.0	67	16.8
					0.122	9.3	18.2	1386.9	76	19.0
					0.121	10.3	17.8	1510.1	85	21.3
6	2.92	2.27	2.24	1.61	0.159	20.3	30.9	3949.0	128	32.0
					0.158	20.6	34.8	4518.7	130	32.5
					0.160	21.7	39.1	5318.7	136	34.0
7	2.77	2.15	2.18	1.52	0.141	15.4	30.2	3290.4	109	27.3
					0.129	13.8	30.0	3214.4	107	26.8
					0.157	15.2	39.1	3794.4	97	24.3
8	2.6	2.04	2.07	1.49	0.184	16.2	44.0	3869.4	88	22.0
					0.185	17.0	45.7	4202.2	92	23.0
					0.148	29.4	39.1	7744.3	198	49.5
9	2.29	1.85	1.93	1.38	0.154	32.9	35.8	7663.3	214	53.5
					0.143	26.6	36.9	6855.4	186	46.5
					0.095	11.0	27.8	3227.7	116	29.0
10	2.61	2.08	2	1.69	0.091	10.5	29.4	3386.7	115	28.7
bifurcation					0.102	8.8	33.9	2914.3	86	21.5
					0.137	11.5	36.2	3036.9	84	21.0
11	2.73	2.22	2.28	1.61	0.178	15.2	42.6	3616.9	85	21.3
					0.110	13.2	46.4	5568.8	120	30.0
					0.153	18.9	31.2	3864.4	124	31.0
12	2.14	1.25	1.16	0.72	0.151	16.5	32.9	3589.6	109	27.3
					0.178	12.8	46.1	3320.7	72	18.0
					0.243	17.5	42.5	3060.1	72	18.0
13	1.97	1.32	1.33	0.77	0.223	11.8	34.4	1820.8	53	13.3
					0.177	15.4	44.2	3845.0	87	21.8
					0.184	14.5	43.5	3434.5	79	19.8
14	1.82	1.25	1.26	0.58	0.199	14.9	53.2	3989.7	75	18.8
					0.146	12.0	43.5	3566.6	82	20.5
					0.151	11.9	37.3	2949.0	79	19.8
15	1.62	1.27	1.27	0.93	0.169	12.7	36.6	2743.5	75	18.8
					0.173	13.4	42.8	3291.9	77	19.3
					0.164	9.8	48.0	2878.0	60	15.0
16	1.57	1.37	1.37	1.09	0.119	4.8	37.7	1507.6	40	10.0

Supplementary Table S10. PET and MRI data from Patient 10

Slice	SUVmax	SUVmean	SUVmedian	SUVmin	FF mean (%)	FF cum (%mm^3)	R2* mean (s^{-1})	R2* cum (mm^3/s)	Voxels (N)	Volume (mm^3)
1	2.33	1.83	1.88	1.08	0.100	9.1	17.7	1611.9	91	22.8
					0.066	6.1	26.9	2500.3	93	23.2
					0.083	9.0	33.0	3562.7	108	27.0
					0.088	9.8	33.1	3706.1	112	28.0
2	2.43	1.81	1.84	1.23	0.093	8.9	33.5	3185.8	95	23.8
					0.094	8.8	31.1	2923.3	94	23.5
					0.116	10.7	38.5	3583.7	93	23.3
3	2.43	2.02	2.07	1.31	0.116	11.1	43.0	4085.0	95	23.8
					0.114	11.3	38.6	3824.3	99	24.8
					0.106	9.4	37.6	3344.1	89	22.3
					0.101	7.7	31.9	2425.4	76	19.0
4	2.44	2.01	1.99	1.25	0.114	8.4	37.4	2764.0	74	18.5
					0.094	8.3	24.8	2184.3	88	22.0
					0.059	5.4	33.2	3054.0	92	23.0
5	2.09	1.72	1.75	1.19	0.072	7.6	50.0	5250.3	105	26.3
					0.154	18.4	55.9	6657.1	119	29.8
					0.148	17.0	55.7	6400.5	115	28.8
					0.137	15.0	63.7	7007.6	110	27.5
6	3.11	1.92	1.91	1.40	0.145	19.1	67.2	8806.3	131	32.8
					0.127	15.7	66.5	8181.5	123	30.8
					0.178	23.4	69.4	9161.2	132	33.0
					0.160	23.1	82.8	11925.5	144	36.0
7	2.46	2.19	2.24	1.53	0.125	17.9	89.1	12830.8	144	36.0
bifurcation					0.067	8.2	48.8	5949.9	122	30.5
					0.090	11.1	39.5	4858.3	123	30.8
8	2.59	1.97	2.05	1.18	0.096	11.2	37.7	4369.5	116	29.0
					0.056	5.5	27.3	2675.9	98	24.5
					0.066	6.3	23.6	2270.2	96	24.0
9	2.53	1.81	1.77	1.41	0.066	6.4	22.8	2214.5	97	24.3
					0.069	6.9	22.9	2262.2	99	24.8
					0.081	7.0	31.3	2725.2	87	21.8

Supplementary Table S11. PET and MRI data from Patient 11

Slice	SUVmax	SUVmean	SUVmedian	SUVmin	FF mean (%)	FF cum (%mm^3)	R2* mean (s^{-1})	R2* cum (mm^3/s)	Voxels (N)	Volume (mm^3)
1	2.26	1.93	1.92	1.55	0.075	6.1	43.9	3589.8	82	20.4
					0.075	7.8	34.5	3589.8	104	26.0
2	2.14	1.69	1.7	1.15	0.072	7.2	34.8	3477.4	100	25.0
					0.078	6.4	56.6	4641.3	82	20.5
					0.126	10.6	64.1	5383.1	84	21.0
					0.173	18.6	63.3	6841.7	108	27.0
3	2.03	1.66	1.66	1.26	0.161	19.8	54.4	6693.0	123	30.8
					0.129	16.5	57.2	7316.9	128	32.0
					0.103	12.5	59.9	7241.9	121	30.3
					0.132	16.6	60.7	7652.3	126	31.5
4	2.33	1.92	1.91	1.33	0.171	20.6	62.4	7553.1	121	30.3
bifurcation					0.136	14.9	60.6	6666.7	110	27.5
					0.150	15.5	72.1	7425.1	103	25.8
5	3.32	2.02	2	1.17	0.152	11.1	70.3	5129.2	73	18.3
					0.109	7.0	79.5	5087.7	64	16.0
					0.082	5.3	56.6	3680.1	65	16.3
					0.058	2.5	34.7	1525.6	44	11.0
6	3.32	1.89	1.81	0.91	0.043	1.7	35.2	1407.0	40	10.0
					0.074	2.4	59.9	1976.0	33	8.3
					0.110	3.0	70.1	1892.9	27	6.8
7	2.55	1.61	1.65	0.91	0.032	0.7	44.5	1024.1	23	5.8
					0.133	4.0	67.8	2033.7	30	7.5
					0.149	5.1	90.3	3068.6	34	8.5
					0.126	3.9	90.6	2807.8	31	7.7
8	2.06	1.67	1.65	1.1	0.172	4.1	86.8	2082.6	24	6.0
					0.137	3.4	66.9	1671.6	25	6.2
					0.060	1.3	53.4	1174.6	22	5.5
9	2.43	1.83	1.84	1.23	0.138	6.9	64.8	3239.4	50	12.5
					0.119	5.9	45.0	2251.3	50	12.5
					0.095	5.8	40.1	2443.3	61	15.3
					0.089	6.6	39.0	2927.4	75	18.8
10	2.73	2.26	2.26	1.41	0.105	7.8	43.0	3184.7	74	18.5
					0.123	9.2	50.8	3812.2	75	18.8
					0.130	9.7	49.2	3691.1	75	18.8
					0.147	8.4	50.5	2879.0	57	14.3

Supplementary Table S12. PET and MRI data from Patient 12

Slice	SUVmax	SUVmean	SUVmedian	SUVmin	FF mean (%)	FF cum (%mm^3)	R2* mean (s^{-1})	R2* cum (mm^3/s)	Voxels (N)	Volume (mm^3)
1	2.67	1.66	1.56	1.04	0.025	0.02	20.9	20.9	1	0.3
					0.236	13.7	30.7	1781.2	58	14.5
					0.205	11.7	33.0	1879.1	57	14.3
					0.238	14.7	44.6	2764.9	62	15.5
2	2.67	1.66	1.56	1.04	0.260	18.7	59.8	4302.6	72	18.0
					0.237	17.1	69.8	5027.1	72	18.0
					0.263	26.3	49.2	4917.3	100	25.0
					0.257	26.5	47.3	4872.8	103	25.8
3	2.94	1.82	1.85	1.04	0.237	25.6	57.1	6162.9	108	27.0
bifurcation					0.251	26.7	80.4	8526.7	106	26.5
					0.210	22.5	75.6	8086.5	107	26.8
4	3.02	1.94	2.00	1.16	0.254	23.1	63.5	5777.8	91	22.8
					0.220	20.5	58.0	5391.3	93	23.3
					0.219	20.4	64.2	5972.9	93	23.3
					0.244	21.0	61.6	5298.8	86	21.5
5	3.50	1.87	1.74	1.13	0.241	20.7	66.2	5689.7	86	21.5
					0.272	23.1	78.4	6667.6	85	21.3
					0.233	17.7	61.7	4691.7	76	19.0
6	2.74	2.22	2.08	1.75	0.213	16.4	58.9	4532.5	77	19.3
					0.272	17.7	50.0	3250.0	65	16.3
					0.237	16.6	46.8	3273.8	70	17.5
					0.259	16.8	47.0	3053.5	65	16.3
7	3.10	2.24	2.08	1.75	0.234	15.4	43.0	2838.0	66	16.5
					0.234	15.7	38.5	2578.8	67	16.8
					0.244	14.9	37.9	2312.6	61	15.3
8	3.24	2.13	2.12	1.34	0.224	16.6	29.8	2207.1	74	18.5
					0.254	17.8	39.6	2775.4	70	17.5
					0.181	7.4	53.9	2208.6	41	10.3
					0.263	11.8	52.3	2354.5	45	11.3
9	3.38	2.08	2.04	1.05	0.267	9.6	62.1	2235.1	36	9.0

Supplementary Table S13. Pearson correlations for target-to-blood pool ratio/fat fraction and for target-to-blood pool ratio/R2* in the subgroup with the shortest time delay (lower tertile) and the longest time delay (upper tertile) between qMRI and PET/MRI.

Days	Correlations TBR/FF	*p*	Correlations TBR/R2*	*p*	Subgroup correlations TBR/FF	Subgroup correlations TBR/R2*	
14	0.16	0.398	-0.23	0.231	-0.196	-0.234	Lower tertile
18	-0.39	0.011	-0.60	0.000			
18	-0.47	0.001	0.20	0.186			
22	-0.09	0.609	-0.31	0.073			
24	-0.12	0.538	0.12	0.506			
26	0.12	0.457	-0.25	0.122			
54	-0.34	0.025	-0.28	0.065			
91	-0.48	0.001	-0.18	0.257			
99	-0.35	0.016	-0.58	0.000	-0.019	-0.094	Upper tertile
104	-0.33	0.026	-0.27	0.077			
160	0.70	0.000	0.14	0.371			
219	-0.09	0.550	0.34	0.021			

In both subgroups there were individuals with either positive or negative correlations, and in both subgroups the correlations were weak regardless of the time delay.

FF, fat fraction; TBR, target-to-blood pool ratio

Supplementary Figure S1. Fat fraction/R2* correlations

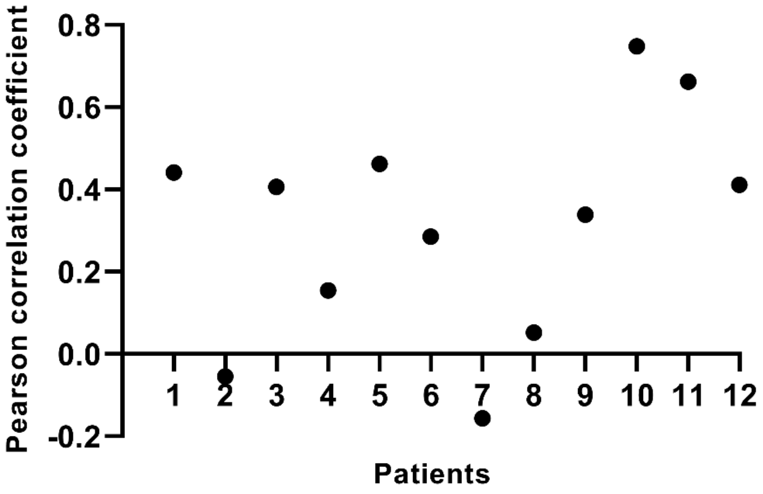

Suppl Fig S1 Pearson correlation coefficients between mean fat fraction and mean R2* for each patient.

FF, fat fraction.

Supplementary Figure S2. Pearson correlations for target-to-blood pool ratio/fat fraction and for target-to-blood pool ratio/R2* sorted by the number of days between qMRI and PET/MRI

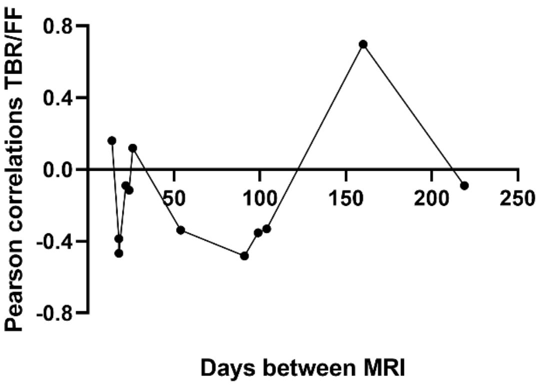

a

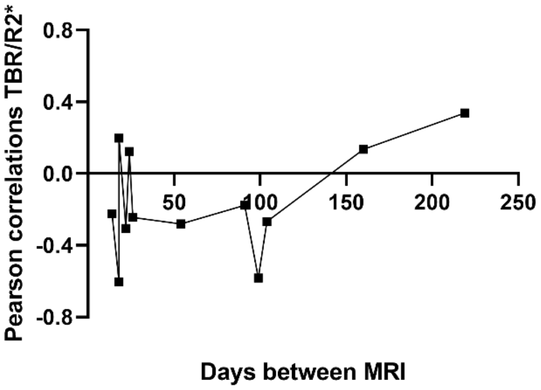

b

Suppl Fig S2 (a) Correlation data for TBR/FF from all 12 patients, sorted by number of days between investigations (b) Correlation data for TBR/R2* from all 12 patients, sorted by number of days between investigations. The plots show no evidence that the correlations between TBR/FF and TBR/R2* were influenced by the length of time between qMRI and PET/MRI.

FF, fat fraction; MRI, Magnetic Resonance Imaging; qMRI, quantitative Magnetic Resonance Imaging; TBR, target-to-blood pool ratio

Supplementary Figure S3. Correlation plots for target-to-blood pool ratio/fat fraction and for target-to-blood pool ratio/R2* in the subgroup with the shortest time delay (lower tertile) and the longest time delay (upper tertile) between qMRI and PET/MRI

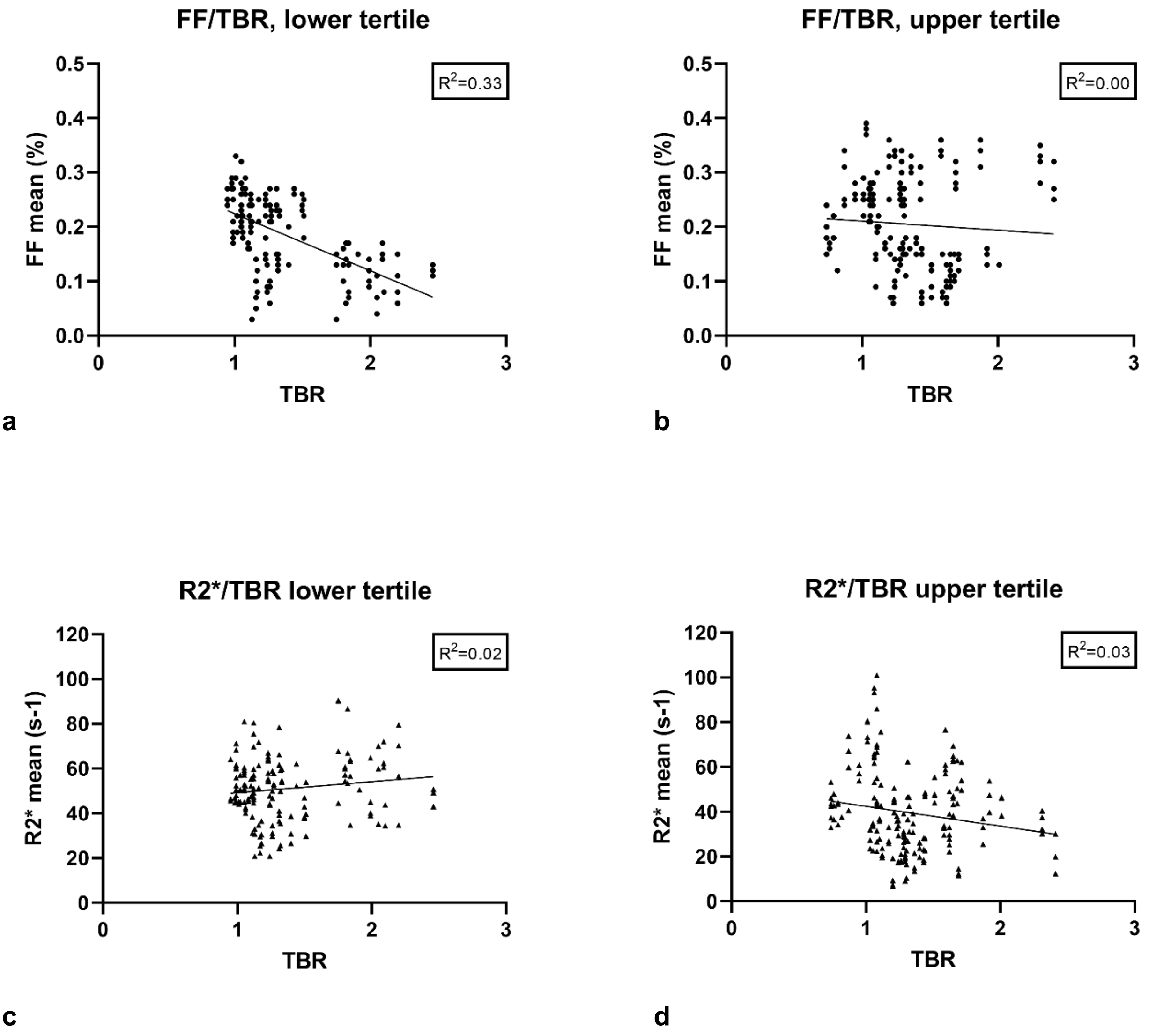

Suppl Fig S3 Linear regressions based on all plaque values from all patients within the lower respectively upper tertile. No positive correlation was found between FF/TBR nor R2*/TBR, regardless of the time delay between qMRI and PET/MRI. Simple linear regression was performed using GraphPad Prism version 9.0.0 for Windows, GraphPad Software, San Diego, California USA, www.graphpad.com.

FF, fat fraction; PET, Positron Emission Tomography, qMRI, quantitative Magnetic Resonance Imaging; TBR, target-to-blood pool ratio

Supplementary Figure S4. Correlation plots for mean FF/R2* and TBR, all patients

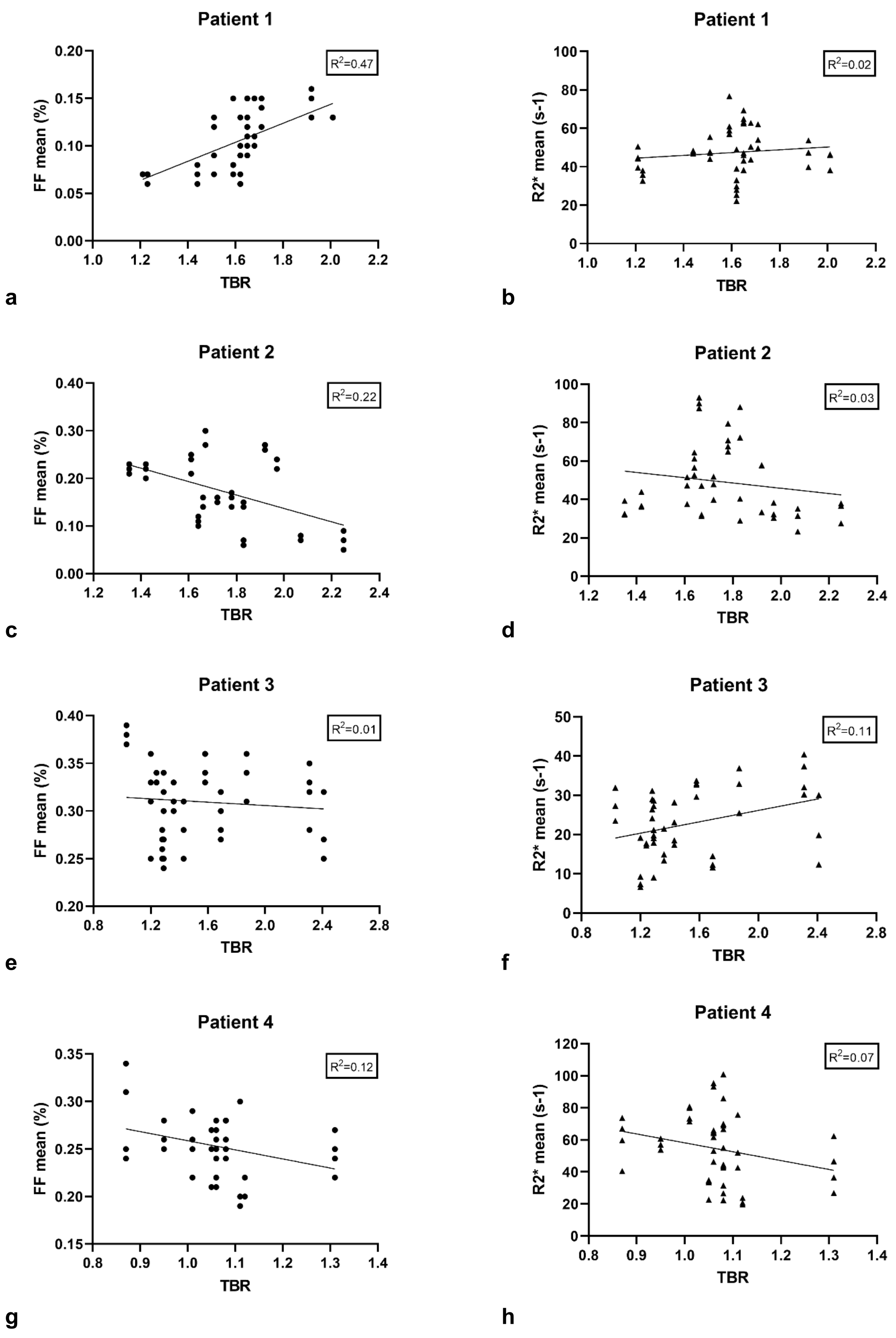

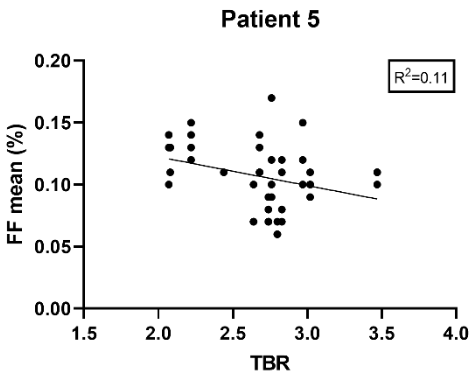
Patient 5
R²=0.11
FF mean (%)
0.00
0.05
0.10
0.15
0.20
1.5
2.0
2.5
3.0
3.5
4.0
TBR
i

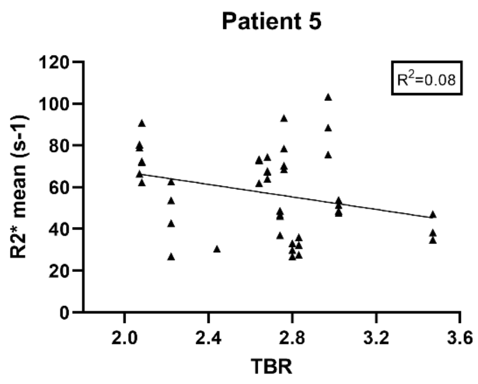
Patient 5
R²=0.08
R2* mean (s-1)
0
20
40
60
80
100
120
2.0
2.4
2.8
3.2
3.6
TBR
j

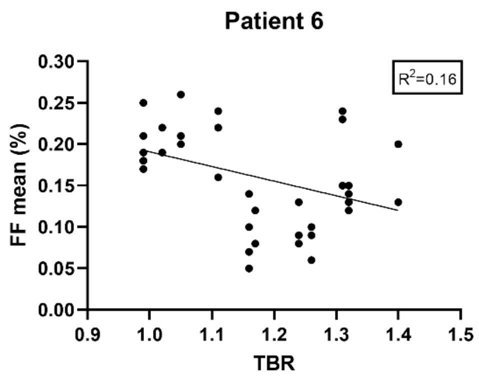
Patient 6
R²=0.16
FF mean (%)
0.00
0.05
0.10
0.15
0.20
0.25
0.30
0.9
1.0
1.1
1.2
1.3
1.4
1.5
TBR
k

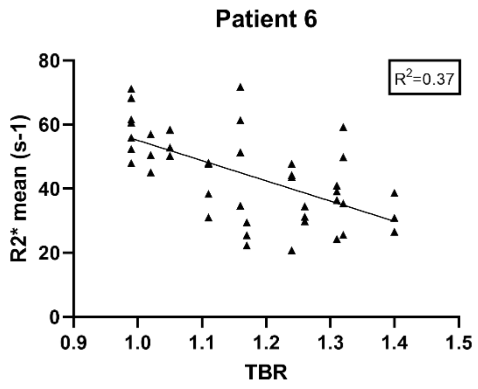
Patient 6
R²=0.37
R2* mean (s-1)
0
20
40
60
80
0.9
1.0
1.1
1.2
1.3
1.4
1.5
TBR
l

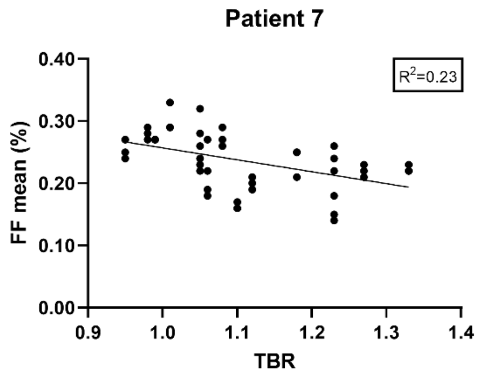
Patient 7
R²=0.23
FF mean (%)
0.00
0.10
0.20
0.30
0.40
0.9
1.0
1.1
1.2
1.3
1.4
TBR
m

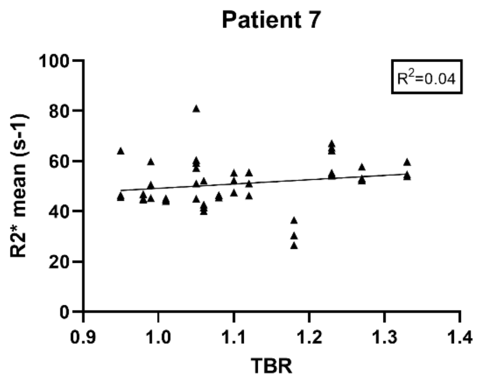
Patient 7
R²=0.04
R2* mean (s-1)
0
20
40
60
80
100
0.9
1.0
1.1
1.2
1.3
1.4
TBR
n

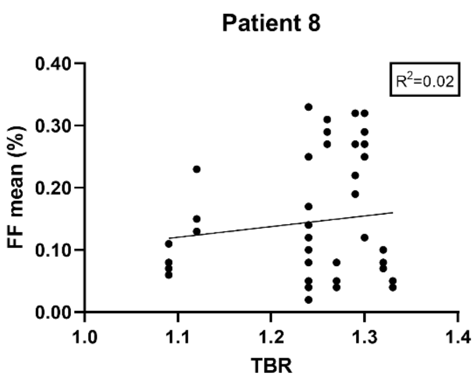
Patient 8
R²=0.02
FF mean (%)
0.00
0.10
0.20
0.30
0.40
1.0
1.1
1.2
1.3
1.4
TBR
o

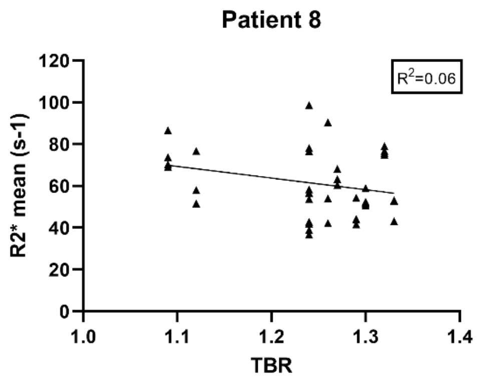
Patient 8
R²=0.06
R2* mean (s-1)
0
20
40
60
80
100
120
1.0
1.1
1.2
1.3
1.4
TBR
p

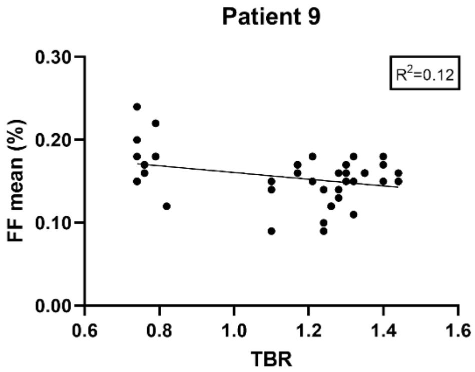
Patient 9
R^2=0.12
FF mean (%)
TBR

q

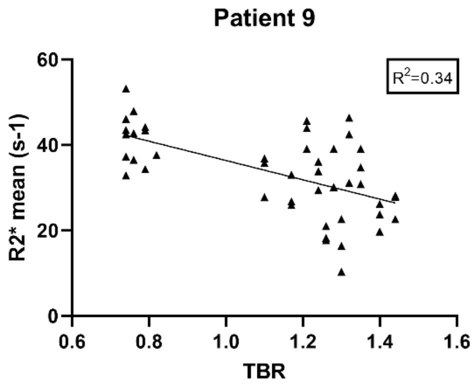
Patient 9
R^2=0.34
R2* mean (s-1)
TBR

r

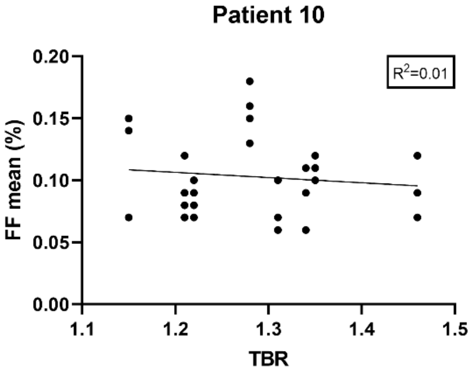
Patient 10
R^2=0.01
FF mean (%)
TBR

s

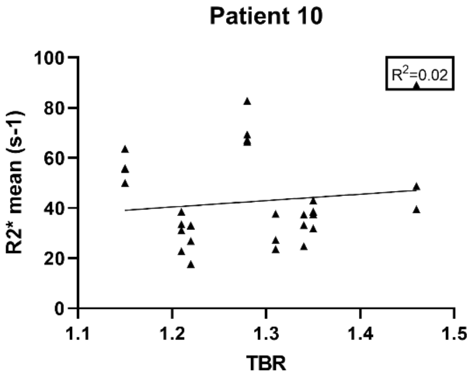
Patient 10
R^2=0.02
R2* mean (s-1)
TBR

t

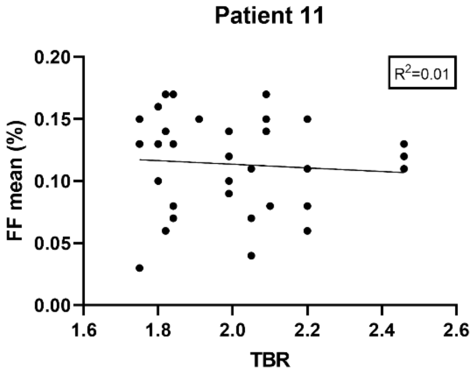
Patient 11
R^2=0.01
FF mean (%)
TBR

u

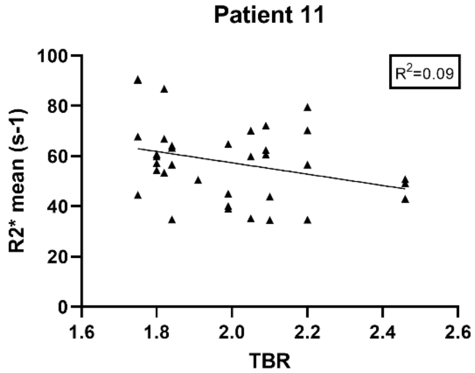
Patient 11
R^2=0.09
R2* mean (s-1)
TBR

v

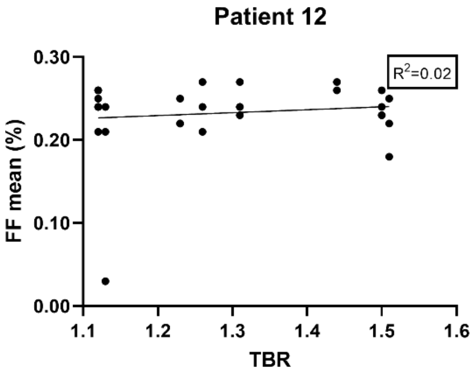
Patient 12
R^2=0.02
FF mean (%)
TBR

x

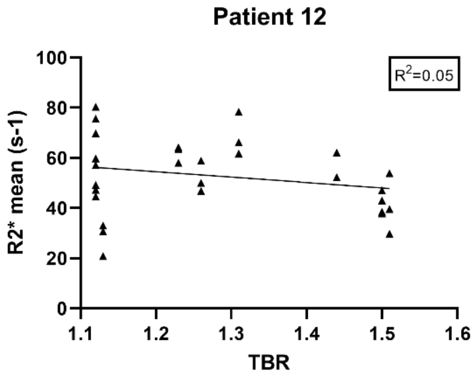
Patient 12
R^2=0.05
R2* mean (s-1)
TBR

y

Suppl Fig S4 Correlation plots in carotid plaques for all 12 patients. Simple linear regression was performed using GraphPad Prism version 9.0.0 for Windows, GraphPad Software, San Diego, California USA, www.graphpad.com

FF, fat fraction; TBR, target-to-blood pool ratio

Supplementary Figure S5. Intraplaque accumulation of ^{18}Fluorodeoxyglucose versus fat fraction and R2*

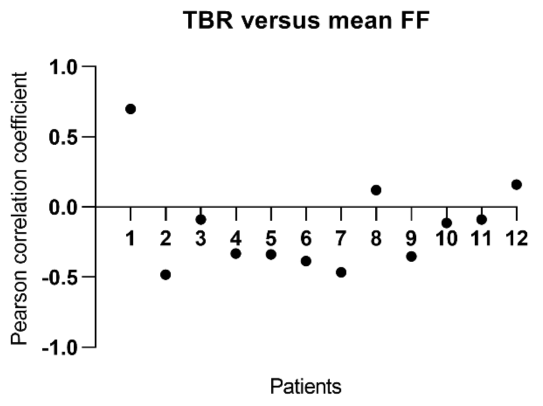

a

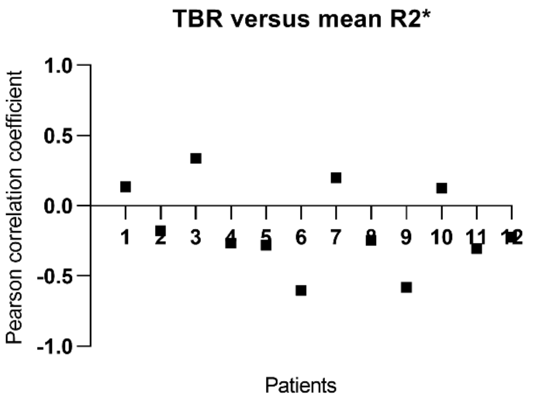

b

Suppl Fig S5 The study number of each patient is listed along the x-axis. There was no statistically significant correlation between FF and TBR for any of the patients. Nor was there any significant correlation between R2*and TBR in this analysis.

FF, fat fraction; TBR, target-to-blood pool ratio
